# Effect of Finerenone on Morbidity and Mortality in CKD

**DOI:** 10.1681/ASN.0000000823

**Published:** 2025-09-12

**Authors:** John W. Ostrominski, Gerasimos Filippatos, Brian L. Claggett, Zi Michael Miao, Akshay S. Desai, Pardeep S. Jhund, Alasdair Henderson, Katja Rohwedder, Meike D. Brinker, Andrea Scalise, Patrick Schloemer, Carolyn S.P. Lam, Michele Senni, Sanjiv J. Shah, Adriaan A. Voors, Faiez Zannad, Peter Rossing, Luis M. Ruilope, Stefan D. Anker, Bertram Pitt, Rajiv Agarwal, John J.V. McMurray, Scott D. Solomon, Muthiah Vaduganathan

**Affiliations:** 1 Cardiovascular Division, Brigham and Women's Hospital, Harvard Medical School, Boston, Massachusetts; 2 School of Medicine, Attikon University Hospital, National and Kapodistrian University of Athens, Athens, Greece; 3 British Heart Foundation Cardiovascular Research Centre, School of Cardiovascular and Metabolic Health, University of Glasgow, Glasgow, Scotland, United Kingdom; 4 Bayer AG, Global Medical Affairs, Berlin, Germany; 5 Bayer AG, Research and Development, Pharmaceuticals, Wuppertal, Germany; 6 Cardiology and Nephrology Clinical Development, Bayer Hispania S.L., Barcelona, Spain; 7 Bayer AG, Research and Development, Pharmaceuticals, Berlin, Germany; 8 National Heart Centre Singapore and Duke-National University of Singapore, Singapore; 9 University of Milano-Bicocca ASST Papa Giovanni XXIII Hospital, Bergamo, Italy; 10 Feinberg Cardiovascular Research Institute, Northwestern University Feinberg School of Medicine, Chicago, Illinois; 11 Groningen Heart Failure Research Institute, University of Groningen, Groningen, The Netherlands; 12 University of Lorraine, Clinical Investigation Centre, Nancy, France; 13 Steno Diabetes Center Copenhagen, Herlev, Denmark; 14 Department of Clinical Medicine, University of Copenhagen, Copenhagen, Denmark; 15 Hospital 12 de Octubre, Madrid, Spain; 16 Department of Cardiology (CVK), German Heart Center Charité, German Centre for Cardiovascular Research (DZHK) partner site Berlin, Charité Universitätsmedizin, Berlin, Germany; 17 Department of Medicine, University of Michigan, Ann Arbor, Michigan; 18 Indiana University School of Medicine, Indianapolis, Indiana

**Keywords:** cardiovascular disease, cardiovascular events, CKD, diabetes, diabetic kidney disease, heart failure

## Abstract

**Key Points:**

- In three trials, finerenone reduced a range of cardiovascular and kidney outcomes in persons with diabetes and CKD.
- Benefits of finerenone were observed across a broad spectrum of glycemia and kidney structure and function.
- In exploratory analyses, cardiovascular benefits of finerenone appeared consistent in CKD populations without diabetes.

**Background:**

Finerenone has been shown to reduce cardiovascular and kidney events in individuals with CKD and type 2 diabetes. Pooling data from all completed outcomes trials evaluating finerenone to date may enhance understanding of its effects on morbidity and mortality in this high-risk population.

**Methods:**

In this prespecified participant-level pooled analysis of the Finerenone in Reducing Kidney Failure and Disease Progression in Diabetic Kidney Disease (FIDELIO-DKD), Finerenone in Reducing Cardiovascular Mortality and Morbidity in Diabetic Kidney Disease (FIGARO-DKD), and Finerenone Trial to Investigate Efficacy and Safety Superior to Placebo in Patients with Heart Failure (FINEARTS-HF) trials (FINE-HEART), FINEARTS-HF participants with type 2 diabetes, CKD, and albuminuria were pooled with persons enrolled in FIDELIO-DKD and FIGARO-DKD. Treatment effects of finerenone versus placebo on cardiovascular events, composite kidney outcomes, all-cause hospitalization, and mortality were evaluated using Cox proportional hazards regression models, stratified by trial and geographic region.

**Results:**

Among 18,991 pooled trial participants, 14,180 (mean age, 65±10 years; 31% female; mean eGFR, 58±22 ml/min per 1.73 m^2^; median urine albumin-to-creatinine ratio (UACR), 478 [interquartile range, 167–1103] mg/g) were included in the analysis. Finerenone reduced cardiovascular death or heart failure hospitalization (hazard ratio, 0.83; 95% confidence interval, 0.75 to 0.93; *P* = 0.001) compared with placebo, without evidence of heterogeneity according to baseline eGFR (*P*_interaction_ = 0.18), UACR (*P*_interaction_ = 0.60), or glycated hemoglobin (*P*_interaction_ = 0.51). Finerenone additionally appeared to reduce cardiovascular death, heart failure hospitalization, major adverse cardiovascular events, new-onset atrial fibrillation, and the composite kidney outcome. Benefits on the composite kidney outcome appeared greater with higher baseline UACR (*P*_interaction_ = 0.04). In sensitivity analyses evaluating a broader range of participants with CKD, finerenone appeared to consistently reduce cardiovascular death or heart failure hospitalization irrespective of (*1*) UACR (*P*_interaction_ = 0.22) when FINEARTS-HF participants with type 2 diabetes and at least moderate-risk CKD were included and (*2*) glycated hemoglobin (*P*_interaction_ = 0.59) when FINEARTS-HF participants with albuminuric CKD but without diabetes were included. Serious adverse events were less common with finerenone versus placebo (34% versus 36%), but hyperkalemia-related permanent treatment discontinuations were more common (2% versus 1%).

**Conclusions:**

In this prespecified FINE-HEART analysis, finerenone reduced cardiovascular, kidney, and mortality events across a wide spectrum of CKD in persons with type 2 diabetes.

**Clinical Trial registry name and registration number:**

PROSPERO, CRD42024570467

## Visual Abstract

**Figure:**
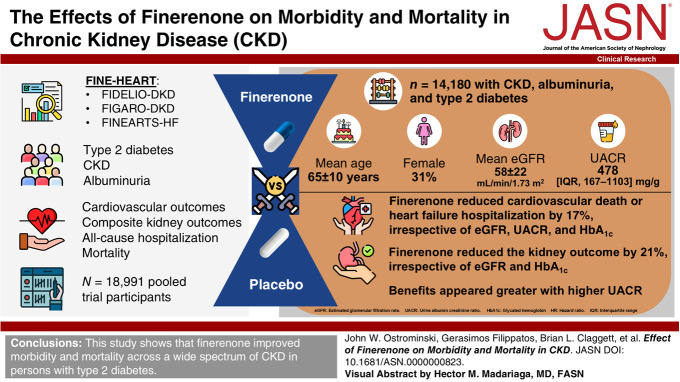

## Introduction

CKD is estimated to affect approximately one in ten persons worldwide^1^ and is a leading but widely underrecognized risk factor for cardiovascular disease and premature mortality.^2–5^ Risks of CKD progression and CKD-related health complications are incrementally higher among persons with concomitant type 2 diabetes,^6^ underscoring the need for therapeutic strategies targeting this increasingly common pathophysiologic intersection.^7^ Finerenone, a nonsteroidal mineralocorticoid receptor antagonist, is recently guideline recommended for persons with type 2 diabetes, CKD, and albuminuria.^8^ However, given the wide spectrum of kidney and cardiovascular risk within this population,^9,10^ further evidence may enhance knowledge of the efficacy and safety of finerenone and inform clinical care.

Thus far, three large-scale trials have assessed the effects of finerenone on cardiovascular and kidney outcomes: Finerenone in Reducing Kidney Failure and Disease Progression in Diabetic Kidney Disease (FIDELIO-DKD),^11^ Finerenone in Reducing Cardiovascular Mortality and Morbidity in Diabetic Kidney Disease (FIGARO-DKD),^12^ and Finerenone Trial to Investigate Efficacy and Safety Superior to Placebo in Patients with Heart Failure (FINEARTS-HF).^13^ Designed to detect treatment effects on kidney events, the FIDELIO-DKD trial included persons with type 2 diabetes and CKD with predominantly very high levels of albuminuria.^14^ Designed to detect treatment effects on cardiovascular outcomes, the FIGARO-DKD selected persons with type 2 diabetes, moderate albuminuria, and a wider range of kidney function.^15^ Designed to detect treatment effects on heart failure outcomes, the FINEARTS-HF trial included individuals with chronic symptomatic heart failure with mildly reduced or preserved ejection fraction, with or without CKD and/or diabetes.^16^ The complementary designs, distinct but overlapping patient populations, and similar end points of these three trials has allowed for a prespecified pooled analysis (FINE-HEART) evaluating the efficacy and safety of finerenone across a broad range of cardiovascular, kidney, and metabolic risks.^17^ However, effects of finerenone on morbidity and mortality across the spectrum of CKD in FINE-HEART have not been explored.

In the prior Finerenone in CKD and Type 2 Diabetes: Combined FIDELIO-DKD and FIGARO-DKD Trial Program Analysis (FIDELITY), finerenone was shown to reduce composite cardiovascular and kidney events by 16% and 23%, respectively.^18^ However, FIDELITY included relatively few persons with established heart failure, which is common in persons with CKD and type 2 diabetes and associated with additive risk of adverse clinical outcomes.^17,19,20^ As such, more robust estimates of the efficacy and safety of finerenone in the growing population of persons with cardiovascular, kidney, and metabolic conditions are needed.^7^ Moreover, the prior FIDELITY analysis was restricted to individuals with albuminuric CKD and type 2 diabetes. As many individuals with CKD do not have moderately or severely increased albuminuria^2,21^ and most individuals with CKD do not have concomitant diabetes,^4,22^ further research may enhance the evidentiary basis for ongoing and further study of finerenone in these broader but relatively understudied populations.

In this prespecified analysis, we evaluated the efficacy and safety of finerenone among FINE-HEART participants with type 2 diabetes and albuminuric CKD, a population with a wider range of cardiovascular risk when compared with prior analyses. Targeting key evidence gaps, we additionally evaluated the cardiovascular and kidney effects of finerenone in sensitivity analyses including (*1*) participants with type 2 diabetes and CKD with urine albumin-to-creatinine ratio (UACR) <30 mg/g and (*2*) participants with CKD and UACR ≥30 mg/g, with or without diabetes.

## Methods

### The Integrated FINE-HEART Program, Patient Population, and Trial Characteristics

The rationale, design, and primary results of FINE-HEART have been previously described.^17^ In brief, FINE-HEART is a prespecified participant-level pooled analysis (prospective PROSPERO registration: CRD42024570467) of three global, multicenter, double-blind, placebo-controlled, randomized clinical trials evaluating the safety and efficacy of finerenone in adults with CKD and type 2 diabetes or heart failure with mildly reduced or preserved ejection fraction, with or without diabetes. Smaller randomized clinical trials evaluating finerenone that were active-controlled (*e.g*., Mineralocorticoid Receptor Antagonist Tolerability Study [NCT01345656] and Mineralocorticoid Receptor Antagonist Tolerability Study - Heart Failure [NCT01807221]), had nonharmonizable primary end point definitions, or had few (<10) cardiovascular events (*e.g*., Mineralocorticoid Receptor Antagonist Tolerability Study - Diabetic Nephropathy [NCT01874431]) were not included. The design, baseline characteristics, and primary results of each of the trials included in FINE-HEART have been previously reported.^11–16,23^

The FIDELIO-DKD and FIGARO-DKD trials enrolled adults (18 years or older) with type 2 diabetes and CKD across 48 countries. FIDELIO-DKD required a UACR of 30 to <300 mg/g, an eGFR of 25 to <60 ml/min per 1.73 m^2^, and a history of diabetic retinopathy or a UACR of 300 to 5000 mg/g and eGFR of 25 to <75 ml/min per 1.73 m^2^. FIGARO-DKD required either a UACR of 30 to <300 mg/g with an eGFR of 25–90 ml/min per 1.73 m^2^ or a UACR of 300 to 5000 mg/g with an eGFR of ≥60 ml/min per 1.73 m^2^. Both trials required a serum potassium of ≤4.8 mEq/L for enrollment. The use of renin-angiotensin system inhibitors and dosing was optimized before randomization during run-in phases (lasting 4–16 weeks) in both trials. Patients with a clinical diagnosis of symptomatic (New York Heart Association class II–IV) heart failure with reduced ejection fraction were excluded, but those with heart failure and higher left ventricular ejection fraction were eligible.

The FINEARTS-HF trial enrolled adults (40 years or older) with symptomatic heart failure with mildly reduced or preserved ejection fraction across 37 countries. Key inclusion criteria included left ventricular ejection fraction ≥40%, elevated natriuretic peptides (adjusted based on atrial fibrillation status and clinical setting of screening), evidence of structural heart disease, and recent diuretic use for ≥30 days. Participants were required to have an eGFR ≥25 ml/min per 1.73 m^2^ and a serum potassium level ≤5.0 mEq/L for enrollment. Participants were eligible for enrollment regardless of type 2 diabetes status, UACR level, and clinical care setting (whether hospitalized, recently hospitalized, or in ambulatory care).

### Trial Procedures

Participants in each of the three trials were randomly allocated to finerenone or placebo. The initial dose of study medication was 10 mg once daily for participants with a baseline eGFR of <60 ml/min per 1.73 m^2^ (FIDELIO-DKD and FIGARO-DKD) or ≤60 ml/min per 1.73 m^2^ (FINEARTS-HF), titrated to a target dose of 20 mg once daily as tolerated. For participants with a baseline eGFR of ≥60 ml/min per 1.73 m^2^ (FIDELIO-DKD and FIGARO-DKD) or >60 ml/min per 1.73 m^2^ (FINEARTS-HF), study medication was initiated at a dose of 20 mg once daily, but further titration to a target dose of 40 mg once daily occurred only in FINEARTS-HF; 20 mg once daily was the target dose in FIDELIO-DKD and FIGARO-DKD, irrespective of baseline eGFR. The trial protocols were approved by ethics committees or institutional review boards at all participating sites, were conducted in accordance with the principles of the Declaration of Helsinki, and all participants provided written informed consent.

### FINE-HEART Pooled Analysis End Points

Individual participant-level data were accessed and pooled with harmonized data elements for baseline characteristics and clinical outcomes.^17^ All participants randomized in each of the three trials were considered for inclusion in the FINE-HEART pooled analysis; only those with critical Good Clinical Practice violations were excluded. As previously reported, 160 randomized participants (*n*=60 in FIDELIO-DKD, *n*=85 in FIGARO-DKD, and *n*=15 in FINEARTS-HF) were prospectively excluded before database lock from all analyses because of critical Good Clinical Practice violations or due to rerandomization of the same participant.^13,18^ In addition, 36 participants in FIDELIO-DKD and FIGARO-DKD were confirmed to have critical Good Clinical Practice violations after database lock.^17^ These 196 participants were excluded from all efficacy and safety analysis in FINE-HEART. All efficacy outcomes were analyzed in randomized patients under intention-to-treat principles, while all safety outcomes were analyzed in randomized patients who had taken ≥1 dose of the study drug.

We analyzed a range of cardiovascular, kidney, and mortality outcomes including cardiovascular death or heart failure hospitalization, cardiovascular death, heart failure hospitalization, major adverse cardiovascular events (a composite of nonfatal myocardial infarction, nonfatal stroke, heart failure hospitalization, and cardiovascular death), new-onset atrial fibrillation, the kidney composite outcome (defined as a sustained decrease in eGFR to ≥50% from baseline, sustained decrease in eGFR to <15 ml/min per 1.73 m^2^, kidney failure, and death due to kidney failure), all-cause hospitalization, and all-cause death. The kidney composite end point inclusive of a sustained decrease in eGFR to ≥57% from baseline (corresponding to a doubling of serum creatinine), sustained decrease in eGFR to <15 ml/min per 1.73  m^2^, kidney failure, and death due to kidney failure, was additionally reported.^17^ The composite of all-cause death or all-cause hospitalization was also reported to describe the total burden of morbidity and mortality.^17^

End points including cardiovascular death were reported as either exclusive (consistent with the primary FINE-HEART analysis) or inclusive (consistent with the prespecified FINE-HEART sensitivity analysis) of deaths due to undetermined causes.^17^ Heart failure hospitalization, new-onset atrial fibrillation, and all deaths were adjudicated by independent clinical end point committees in each of the trials included in this analysis. Select treatment-emergent adverse events related to hyperkalemia, AKI, hypotension, and gynecomastia were also reported in the pooled population.

### Statistical Analysis

Participants in the FIDELIO-DKD and FIGARO-DKD trials, in addition to FINEARTS-HF participants with type 2 diabetes, eGFR >25 ml/min per 1.73 m^2^, and UACR >30 mg/g, were included in this pooled analysis. Baseline characteristics were compared according to randomized treatment using *t* tests or Wilcoxon rank-sum tests for comparison of continuous variables, as appropriate, and chi-squared tests for comparison of categorical variables. All end points were analyzed as time-to-first end points using Cox proportional hazards models, stratified by geographic region and trial (account for differences in trial design). As prespecified in the FINE-HEART statistical analysis plan,^17^ all treatment effect estimates were presented as unadjusted hazard ratios (HR) with 95% confidence intervals (CI). Outcome incidence rates (by treatment arm) and absolute rate differences (per 1000 person-years) were estimated through Poisson regression. Multivariable Cox proportional hazards regression models and subdistribution hazard regression (Fine–Gray) models (adjusted for trial and geographic region) were additionally constructed to evaluate for potential confounding and competing risks of all-cause death, respectively.

Selected end points were graphically displayed by treatment arm using Kaplan–Meier methods, and treatment effects of finerenone versus placebo on cardiovascular death or heart failure hospitalization and the composite kidney outcome (inclusive of ≥50% eGFR decrease) were evaluated across key subgroups. To assess for treatment effect modification according to baseline eGFR, UACR, and glycated hemoglobin (HbA_1c_), incidence rates (per 1000 person-years) of cardiovascular death or heart failure hospitalization and the composite kidney outcomes were estimated by treatment arm through Poisson regression. To allow for potentially nonlinear relationships, model output was displayed using restricted cubic splines; the number of knots was selected to minimize the Akaike information criterion. Dedicated Cox proportional hazards regression models were additionally constructed to assess for treatment effect modification by baseline eGFR, UACR, and HbA_1c_.

Two sensitivity analyses were performed. First, to explore the treatment effects of finerenone on key end points across a broader range of kidney risk, FINEARTS-HF participants with type 2 diabetes and at least moderate-risk CKD (eGFR ≤60 ml/min per 1.73 m^2^ or UACR ≥30 mg/g) were combined with participants with the FIDELIO-DKD and FIGARO-DKD trials. Second, to ascertain the effect of finerenone across a broader range of baseline glycemia, FINEARTS-HF participants with and without diabetes, eGFR >25 ml/min per 1.73 m^2^, and UACR >30 mg/g were combined with participants from the FIDELIO-DKD and FIGARO-DKD trials. Statistical analyses were conducted using Stata, version 18.5 (StataCorp, LLC; College Station, TX), with two-sided *P* values < 0.05 considered statistically significant. Consistent with previous studies,^17,18^ no adjustment for multiplicity was performed in this pooled analysis.

## Results

### Patient Population

Of 18,991 pooled trial participants, 4811 FINEARTS-HF participants (Supplemental Table 1) were excluded due to missing data for baseline UACR (*n*=204) or not having the metabolic (type 2 diabetes) and kidney (eGFR >25 ml/min per 1.73 m^2^ and UACR >30 mg/g) criteria for inclusion in the analysis (*n*=4607). As such, 14,180 (mean age, 65±10 years; 31% female; mean eGFR, 58±22 ml/min per 1.73 m^2^; median UACR, 478 [interquartile range, 167–1102] mg/g) were included in the analysis. Baseline characteristics and background pharmacotherapies were generally well-balanced between treatment arms (Table 1). Most FINE-HEART participants with CKD and type 2 diabetes included in this analysis had either high or very high kidney risk (88%) at baseline; 12% and 1% had moderately higher and low kidney risk at baseline, respectively (Supplemental Figure 1).

**Table 1:** Baseline characteristics of FINE-HEART participants with CKD and type 2 diabetes, by treatment arm

Characteristic	Overall (*n*=14,180)	Finerenone (*n*=7094)	Placebo (*n*=7086)
Age, yr	65±10	65±10	65±10
Female, *n* (%)	4417 (31)	2266 (32)	2151 (30)
**Race^a^, *n* (%)**			
Asian	3086 (22)	1526 (22)	1560 (22)
Black	535 (4)	259 (4)	276 (4)
Other	773 (6)	402 (6)	371 (5)
White	9786 (69)	4899 (69)	4887 (69)
**Region, *n* (%)**			
Asia	2862 (20)	1425 (20)	1437 (20)
Eastern Europe	3738 (26)	1894 (27)	1844 (26)
Latin America	1579 (11)	794 (11)	785 (11)
North America	2167 (15)	1080 (15)	1087 (15)
Western Europe, Oceania, and others	3834 (27)	1893 (27)	1941 (27)
**Trial, *n* (%)**			
FIDELIO-DKD	5662 (40)	2824 (40)	2838 (40)
FIGARO-DKD	7328 (52)	3674 (52)	3654 (52)
FINEARTS-HF	1190 (8)	588 (8)	602 (9)
BMI, kg/m^2^	31±6	31±6	31±6
Waist circumference, cm	107±15	107±15	107±15
Waist-hip ratio	1.0±0.1	1.0±0.1	1.0±0.1
Systolic BP, mm Hg	136±14	136±14	136±14
Potassium, mEq/L	4.4±0.4	4.4±0.4	4.4±0.5
HbA_1c_, %	7.7±1.4	7.7±1.4	7.7±1.4
eGFR, ml/min per 1.73 m^2^	58±22	57±21	58±22
**eGFR category,** **ml/min per 1.73 m**^**2**^**, *n* (%)**			
<25	162 (1)	81 (1)	81 (1)
25 to <45	4605 (33)	2302 (33)	2303 (33)
45 to <60	3762 (27)	1880 (27)	1882 (27)
≥60	5648 (40)	2822 (40)	2826 (40)
UACR, mg/g	478 (167–1102)	479 (168–1093)	477 (166–1117)
**Baseline UACR category, mg/g, *n*** (**%)**			
<30	230 (2)	120 (2)	110 (2)
30 to <300	4887 (35)	2458 (35)	2429 (34)
≥300	9058 (64)	4506 (64)	4552 (64)
History of heart failure, *n* (%)	2197 (16)	1073 (15)	1124 (16)
History of atrial fibrillation, *n* (%)	1718 (12)	864 (12)	854 (12)
Atrial fibrillation on electrocardiogram, *n* (%)	984 (7)	499 (7)	485 (7)
Duration of diabetes, yr	15±9	15±9	15±9
**Duration of diabetes category, yr, *n* (%)**			
<5	1612 (11)	779 (11)	833 (12)
≥5 to <10	2538 (18)	1288 (18)	1250 (18)
≥10 to <15	3058 (22)	1558 (22)	1500 (21)
≥15 to <20	2974 (21)	1441 (20)	1533 (22)
≥20 to <25	2026 (14)	1034 (15)	992 (14)
≥25	1941 (14)	969 (14)	972 (14)
**Background medication use, *n* (%)**			
Diuretics	7875 (56)	3898 (55)	3977 (56)
ACEi	5447 (38)	2708 (38)	2739 (39)
ARB	8357 (59)	4211 (59)	4146 (58)
ARNI	127 (1)	61 (1)	66 (1)
Aspirin	6801 (48)	3407 (48)	3394 (48)
Statin	10,279 (73)	5092 (72)	5187 (73)
SGLT2i	1194 (8)	599 (9)	595 (8)
GLP-1 RA	1045 (7)	542 (8)	503 (7)
Potassium-lowering therapies^b^	189 (1)	98 (1)	91 (1)

Values reported as *n* (%), mean±SD, or median (interquartile range); ACEi, angiotensin-converting enzyme inhibitors; ARB, angiotensin receptor blocker; ARNI, angiotensin receptor-neprilysin inhibitor; BMI, body mass index; FIDELIO-DKD, Finerenone in Reducing Kidney Failure and Disease Progression in Diabetic Kidney Disease; FIGARO-DKD, Finerenone in Reducing Cardiovascular Mortality and Morbidity in Diabetic Kidney Disease; FINEARTS-HF, Finerenone Trial to Investigate Efficacy and Safety Superior to Placebo in Patients with Heart Failure; GLP-1 RA, glucagon-like peptide-1 receptor agonist; HbA_1c_, glycated hemoglobin; SGLT2i, sodium-glucose cotransporter 2 inhibitor; UACR, urine albumin-to-creatinine ratio.

a Represents self-reported race. Participants electing to not disclose race or who self-identified as multiple races are included in the “Other” category for descriptive purposes.

b Includes patiromer, sodium polystyrene sulfonate, and calcium polystyrene sulfonate.

Compared with FIDELITY participants (*n*=12,990), FINEARTS-HF participants with CKD and type 2 diabetes (*n*=1190) were older, were more likely to be female and White, and had lower baseline systolic BP and HbA_1c_ (Supplemental Table 2). However, FINEARTS-HF participants more often had atrial fibrillation and baseline use of diuretics, sodium-glucose cotransporter 2 inhibitors, and glucagon-like peptide-1 receptor agonists. Although the mean eGFR was similar, FINEARTS-HF participants had lower median UACR (132 mg/g versus 515 mg/g) at baseline.

### Treatment Effects of Finerenone on Clinical Outcomes

**Figure 1:**
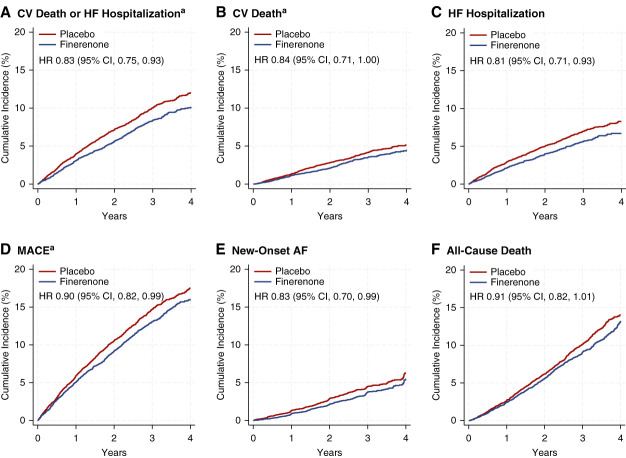
**Cumulative incidence of cardiovascular and mortality outcomes, by treatment arm.** Exclusive of death due to undetermined causes. Figure displays the cumulative incidence of selected time-to-first cardiovascular and mortality outcomes. (A) Cardiovascular death or heart failure hospitalization; (B) cardiovascular death; (C) heart failure hospitalization; (D) major adverse cardiovascular events; (E) new-onset atrial fibrillation; (F) all-cause death. Major adverse cardiovascular events reflects the composite of nonfatal myocardial infarction, nonfatal stroke, heart failure hospitalization, and cardiovascular death. AF, atrial fibrillation; CI, confidence interval; CV, cardiovascular; HF, heart failure; HR, hazard ratio; MACE, major adverse cardiovascular events. ^a^Exclusive of death due to undetermined causes.

**Figure 2:**
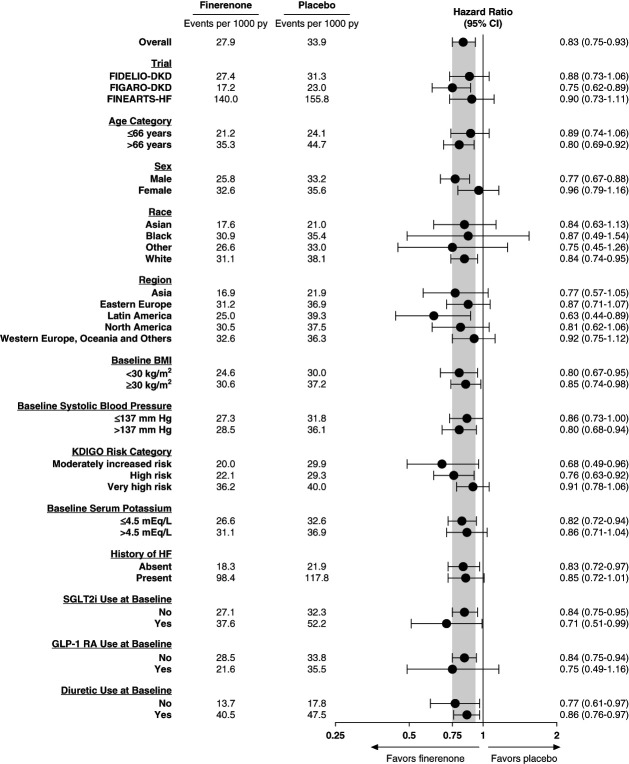
**Effect of finerenone on cardiovascular death or HF hospitalization in FINE-HEART participants with CKD and type 2 diabetes, overall and in key subgroups.** Forest plot shows treatment effects of finerenone versus placebo on cardiovascular death or heart failure hospitalization, overall and in key subgroups. BMI, body mass index; FIDELIO-DKD, Finerenone in Reducing Kidney Failure and Disease Progression in Diabetic Kidney Disease; FIGARO-DKD, Finerenone in Reducing Cardiovascular Mortality and Morbidity in Diabetic Kidney Disease; FINEARTS-HF, Finerenone Trial to Investigate Efficacy and Safety Superior to Placebo in Patients with Heart Failure; GLP-1 RA, glucagon-like peptide-1 receptor agonist; KDIGO, Kidney Disease Improving Global Outcomes; py, person-years; SGLT2i, sodium-glucose cotransporter 2 inhibitor.

**Figure 3:**
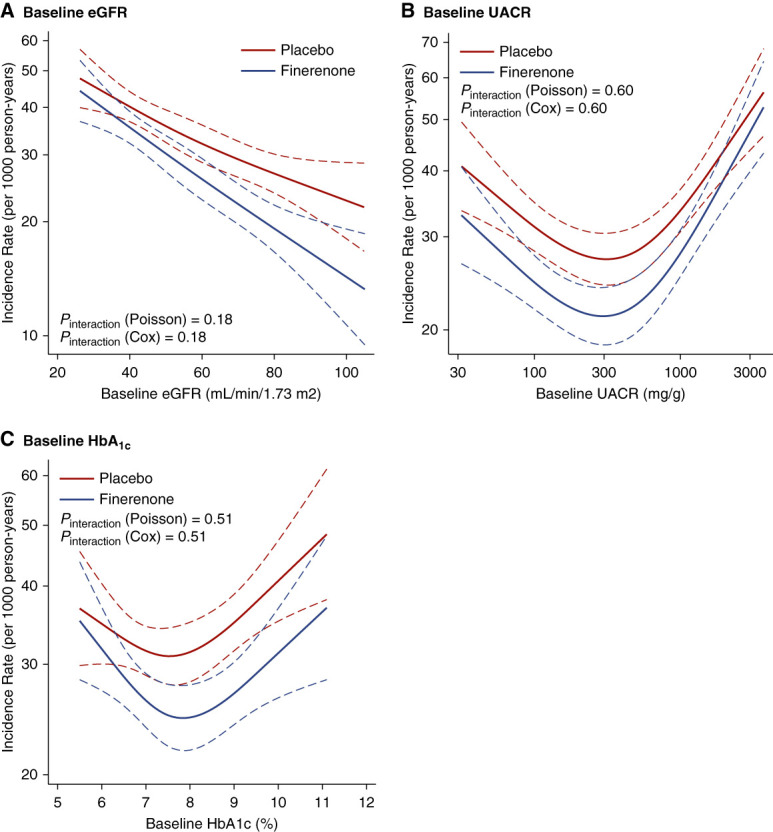
**Treatment effects of finerenone versus placebo on cardiovascular death or HF hospitalization among FINE-HEART participants with CKD and type 2 diabetes, according to baseline eGFR, UACR, and HbA**_**1c**_**.** Figure shows the incidence rate per 1000 py (and 95% CI), estimated through Poisson regression, of cardiovascular death or heart failure by treatment arm, according to baseline eGFR (A), UACR (B), and HbA_1c_ (C). Treatment effect modification evaluated through Poisson regression and Cox proportional hazards regression, in both cases with restricted cubic splines with three knots. Cardiovascular death is exclusive of deaths with undetermined causes. Comparisons reflect use of pooled individual-level data. HbA_1c_, glycated hemoglobin; UACR, urine albumin-to-creatinine ratio.

Over a median follow-up of 3.0 years (interquartile range, 2.3–3.7), cardiovascular death or heart failure hospitalization occurred in 576 (8%) participants in the finerenone arm and in 694 (10%) participants in the placebo arm (HR, 0.83; 95% CI, 0.75 to 0.93; *P* = 0.001) (Figure 1 and Table 2). These effects corresponded to a number-needed-to-treat of 60 to prevent one cardiovascular death or heart failure hospitalization. Consistent effects of finerenone on cardiovascular death or heart failure hospitalization were observed across trials and other key subgroups (Figure 2) and across the range of baseline eGFR (*P*_interaction_ = 0.18), UACR (*P*_interaction_ = 0.60), and HbA_1c_ (*P*_interaction_ = 0.51) (Figure 3).

**Table 2:** Treatment effects of finerenone versus placebo on morbidity and mortality in FINE-HEART participants with CKD and type 2 diabetes

Outcomes	Finerenone (*n*=7094)		Placebo (*n*=7086)		Finerenone versus Placebo	Rate Difference per 1000 py (95% CI)
	# With Event (%)	Incidence Rate (per 1000 py)	# With Event (%)	Incidence Rate (per 1000 py)	HR (95% CI)	
**Cardiovascular events** ^a^						
Cardiovascular death or heart failure hospitalization	576 (8)	27.9	694 (10)	33.9	0.83 (0.75 to 0.93)	−6.0 (−9.4 to −2.6)
Cardiovascular death	242 (3)	11.4	288 (4)	13.6	0.84 (0.71 to 1.00)	−2.2 (−4.3 to −0.1)
Heart failure hospitalization	383 (5)	18.6	475 (7)	23.2	0.81 (0.71 to 0.93)	−4.6 (−7.4 to −1.8)
Major adverse cardiovascular events	916 (13)	45.6	1017 (14)	51.2	0.90 (0.82 to 0.99)	−5.5 (−9.8 to −1.2)
New-onset atrial fibrillation	227 (4)^b^	12.3	273 (4)^b^	14.8	0.83 (0.70 to 0.99)	−2.5 (−4.9 to −0.2)
**Cardiovascular events** ^c^						
Cardiovascular death or heart failure hospitalization	723 (10)	35.0	844 (12)	41.2	0.86 (0.78 to 0.95)	−6.2 (−10.0 to −2.4)
Cardiovascular death	409 (6)	19.3	467 (7)	22.1	0.88 (0.77 to 1.00)	−2.8 (−5.5 to −0.1)
Major adverse cardiovascular events	1039 (15)	51.8	1159 (16)	58.3	0.90 (0.82 to 0.97)	−6.5 (−11.1 to −1.9)
**Kidney outcomes**						
Composite kidney outcome (eGFR ≥50%)^d^	514 (7)	26.7	645 (9)	33.7	0.79 (0.70 to 0.88)	−7.0 (−10.5 to −3.5)
Composite kidney outcome (eGFR ≥57%)^e^	373 (5)	19.2	477 (7)	24.7	0.78 (0.69 to 0.89)	−5.5 (−8.4 to −2.5)
**All-cause morbidity and mortality**						
All-cause death	688 (10)	32.4	757 (11)	35.8	0.91 (0.82 to 1.01)	−3.4 (−6.9 to 0.1)
All-cause hospitalization	3165 (45)	201.4	3260 (46)	209.4	0.97 (0.92 to 1.01)	−8.1 (−18.1 to 2.0)
All-cause death or all-cause hospitalization	3300 (47)	209.9	3426 (48)	220.1	0.96 (0.91 to 1.00)	−10.2 (−20.4 to 0.1)

CI, confidence interval; HR, hazard ratio; py, person-years.

a End points inclusive of cardiovascular death exclusive of deaths with undetermined causes.

b Percentage displayed out of participants without atrial fibrillation at baseline.

c End points inclusive of cardiovascular death inclusive of deaths with undetermined causes.

d Reflects time to first sustained decrease in eGFR to ≥50% from baseline, sustained decrease in eGFR to <15 ml/min per 1.73 m^2^, kidney failure, and death due to kidney failure.

e Reflects time to first sustained decrease in eGFR to ≥57% from baseline, sustained decrease in eGFR to <15  ml/min per 1.73 m^2^, kidney failure, and death due to kidney failure. Major adverse cardiovascular events reflects the composite of nonfatal myocardial infarction, nonfatal stroke, heart failure hospitalization, or cardiovascular death.

Finerenone additionally reduced the risk of heart failure hospitalization (HR, 0.81; 95% CI, 0.71 to 0.93; *P* = 0.002)—these effects were consistent irrespective of history of heart failure at baseline (*P*_interaction_ = 0.81)—and new-onset atrial fibrillation (HR, 0.83; 95% CI, 0.70 to 0.99; *P* = 0.042) compared with placebo (Figure 1). Finerenone additionally appeared to reduce cardiovascular death (HR, 0.84; 95% CI, 0.71 to 1.00; *P* = 0.05), effects that were nominally statistically significant only when deaths due to undetermined causes were included (HR, 0.88; 95% CI, 0.77 to 1.00; *P* = 0.049). Finerenone additionally reduced major cardiovascular events (Figure 1) but did not statistically significantly reduce all-cause death, all-cause hospitalization, or the exploratory composite outcome of all-cause death or all-cause hospitalization (Table 2). Similar findings for all end points were observed after covariate adjustment (Supplemental Table 3) and after considering competing risk of all-cause death (Supplemental Table 4).

**Figure 4:**
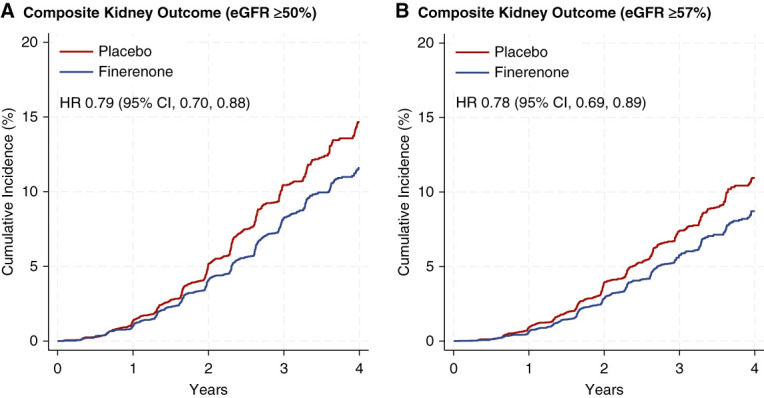
**Cumulative incidence of composite kidney outcomes, by treatment arm.** Figure displays the cumulative incidence composite kidney outcomes: (A) eGFR ≥50%; (B) eGFR ≥57%.

**Figure 5:**
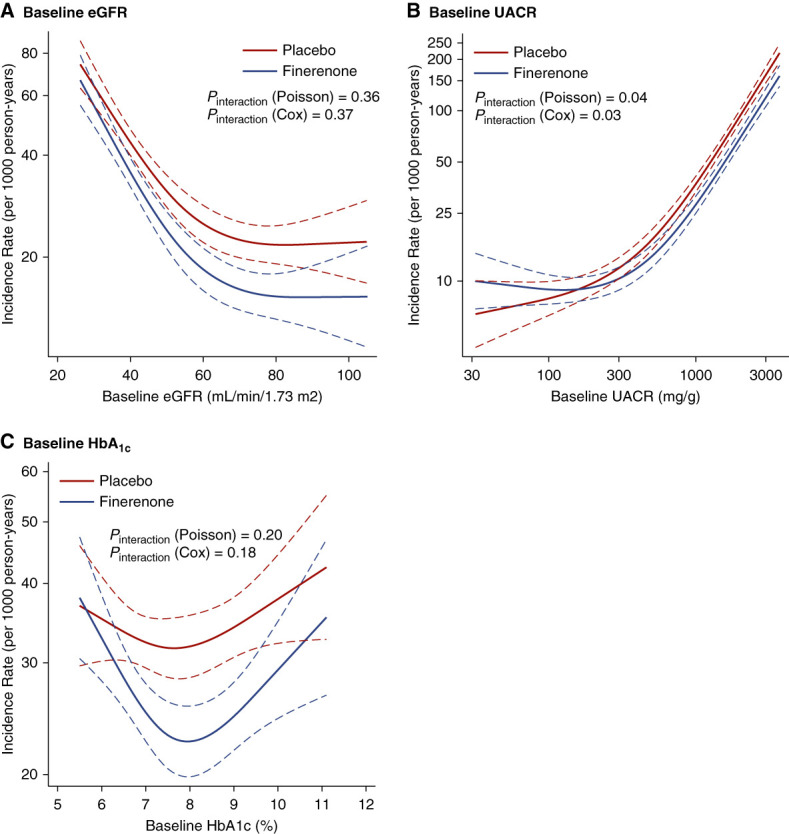
**Treatment effects of finerenone versus placebo on the composite kidney outcome among FINE-HEART participants with CKD and type 2 diabetes, according to baseline eGFR, UACR, and HbA**_**1c**_**.** Figure shows the incidence rate per 1000 py (and 95% CI), estimated through Poisson regression, of the composite kidney outcome (sustained decrease in eGFR to ≥50% from baseline, sustained decrease in eGFR to <15 ml/min per 1.73 m^2^, kidney failure, and death due to kidney failure), according to (A) baseline eGFR, (B) UACR, and (C) HbA_1c_. Treatment effect modification evaluated through Poisson regression and Cox proportional hazards regression, in both cases with restricted cubic splines with three knots. Comparisons reflect use of pooled individual-level data.

In addition, finerenone reduced the composite kidney outcome inclusive of ≥50% eGFR decrease by 21% (HR, 0.79; 95% CI, 0.70 to 0.88; *P* < 0.001) (Figure 4 and Table 2). This reduction corresponded to a number-needed-to-treat of 54 to prevent one kidney event. Similar findings were observed for the composite kidney end point inclusive of ≥57% eGFR decrease (Table 2 and Supplemental Figure 2). Overall kidney effects of finerenone appeared to be driven by FIDELIO-DKD and FIGARO-DKD but were consistent across key subgroups (Supplemental Figure 3). Baseline eGFR did not appear to modify treatment effects of finerenone versus placebo on either composite kidney outcome (Figure 5). However, there was evidence of heterogeneity according to baseline UACR, wherein greater benefits of finerenone on the composite kidney outcome inclusive of ≥50% eGFR decrease were observed at higher levels of UACR (*P*_interaction_ = 0.04) (Figure 5). A similar shape of association was observed for the composite kidney outcome inclusive of ≥57% eGFR decrease but was without strong evidence of effect modification by UACR (*P*_interaction_ = 0.14) (Figure 5).

Broadly, similar effects of finerenone on cardiovascular, kidney, and mortality outcomes were observed in the sensitivity analysis (*n* = 14,700) including all FINEARTS-HF participants with type 2 diabetes and at least moderate-risk CKD (Supplemental Table 5), among whom 737 (5.0%) had UACR <30 mg/g. However, no evidence of effect modification according to eGFR or UACR was observed for the composite cardiovascular outcome or the composite kidney outcome when this broader range of UACR was evaluated (Supplemental Figure 4). In the second sensitivity analysis (*n*=15,212) inclusive of FINEARTS-HF participants with albuminuric CKD with or without type 2 diabetes, marginally stronger treatment effects of finerenone versus placebo on all end points were observed (Supplemental Table 6). In particular, finerenone appeared to statistically significantly reduce cardiovascular death (HR, 0.85; 95% CI, 0.73 to 0.99; *P* = 0.041), irrespective of whether deaths due to undetermined causes were included, and reduce all-cause death (HR, 0.90; 95% CI, 0.82 to 1.00; *P* = 0.023). Furthermore, baseline HbA_1c_ did not appear to modify treatment benefits of finerenone on cardiovascular death or heart failure hospitalization (*P*_interaction_ = 0.59) or the composite kidney outcome (*P*_interaction_ = 0.14) (Supplemental Figure 5).

### Safety Events

The incidence of any serious adverse event was lower with finerenone (34%) compared with placebo (36%) (Table 3). Laboratory-defined hyperkalemia (any serum potassium >5.5 mEq/L) was increased (18% versus 8%), whereas laboratory-defined hypokalemia was reduced (5% versus 10%), with finerenone versus placebo. Hyperkalemia-related treatment discontinuations (2% versus 1%) and investigator-reported hyperkalemia-related hospitalizations (1% versus 0.2%) were also increased with finerenone compared with placebo, but absolute event rates were low. Namely, the absolute risk difference between the finerenone and placebo arms corresponded to a number-needed-to-treat of 131 to cause one hyperkalemia-related hospitalization. There were no deaths related to hyperkalemia in either treatment arm. Investigator-reported AKI, AKI leading to hospitalization, and AKI leading to treatment discontinuation were similar between treatment arms.

**Table 3:** Safety outcomes of FINE-HEART participants with CKD and type 2 diabetes

Safety Outcomes	Finerenone (*n*=7076)	Placebo (*n*=7076)
Any serious adverse event, *n* (%)	2402 (34)	2516 (36)
Any adverse event leading to treatment discontinuation, *n* (%)	441 (6)	373 (5)
Any potassium >5.5 mEq/L^a^, *n* (%)	1232 (18)	566 (8)
Any potassium >6.0 mEq/L^a^, *n* (%)	252 (4)	101 (1)
Any potassium <3.5 mEq/L^a^, *n* (%)	338 (5)	711 (10)
Hyperkalemia^b^, *n* (%)	1013 (14)	512 (7)
Hyperkalemia leading to treatment discontinuation, *n* (%)	114 (2)	41 (1)
Hyperkalemia leading to hospitalization^b^, *n* (%)	69 (1)	15 (0.2)
AKI^b^, *n* (%)	266 (4)	272 (4)
AKI leading to treatment discontinuation, *n* (%)	14 (0.2)	11 (0.2)
AKI leading to hospitalization, *n* (%)	110 (2)	100 (1)
Any systolic BP <100 mm Hg, *n* (%)	587 (8)	341 (5)
Gynecomastia or breast hyperplasia, *n* (%)	10 (0.1)	16 (0.2)

Treatment-emergent adverse events are defined as any adverse event occurring in any patient who has received at least one dose of study drug and within 3 days of permanent discontinuation. There were no instances of death due to hyperkalemia.

a Based on central laboratory measurements of potassium levels.

b Based on investigator-reported adverse events.

Incidences of hyperkalemia and hypokalemia according to baseline eGFR category are presented in Supplemental Table 7. Irrespective of assigned treatment, incidences of hyperkalemia increased with lower baseline eGFR, while incidences of hypokalemia were similar across eGFR categories. Compared with placebo, finerenone was generally associated with higher incidences of hyperkalemia, and lower incidences of hypokalemia, in all eGFR categories. However, incidences of any serum potassium >6.0 mEq/L were low, including among participants with eGFR <30 ml/min per 1.73 m^2^ (6% versus 2% of those treated with finerenone and placebo, respectively), with a similar pattern observed for hyperkalemia-related hospitalization (3% versus 1% of those treated with finerenone and placebo, respectively).

## Discussion

In this prespecified pooled analysis of three outcomes trials evaluating finerenone in individuals with CKD and type 2 diabetes, finerenone reduced a wide spectrum of adverse clinical outcomes in persons at high risk of CKD progression and cardiovascular events. Across the full FINE-HEART subpopulation with CKD and type 2 diabetes, finerenone reduced cardiovascular death or heart failure hospitalization by 17%, and reduced the composite kidney outcome by 23%. Finerenone additionally appeared to reduce heart failure hospitalization, cardiovascular death, major adverse cardiovascular events, and new-onset atrial fibrillation. Benefits on finerenone on cardiovascular death or heart failure hospitalization were consistent across all key subgroups, including participants taking sodium-glucose cotransporter 2 inhibitors or glucagon-like peptide-1 receptor agonists at baseline, and across a broad range of eGFR, UACR, and HbA_1c_. Taken together, these findings provide further support for the use of finerenone to improve morbidity and mortality in persons with CKD and type 2 diabetes.

Findings from this FINE-HEART analysis support and extend those of FIDELITY.^18^ Namely, inclusion of individuals from FINEARTS-HF with CKD and type 2 diabetes improved representation of older adults, women, and individuals with established cardiovascular disease when compared with the pooled FIDELITY population, incrementally extending generalizability into these important but previously understudied groups with unique risk profiles and clinical priorities. Moreover, the FINEARTS-HF participants included in this analysis had lower baseline HbA_1c_ levels, average diabetes duration, and albuminuria. Although collectively indicating lower short-term risk of CKD progression when compared with FIDELITY,^24^ these features also reflect earlier stages of type 2 diabetes and CKD that may be more broadly generalizable to community-dwelling individuals with these conditions.^2,10,25^ Critically, even after inclusion of individuals from FINEARTS-HF with lower kidney risk, finerenone significantly reduced cardiovascular and kidney events when compared with placebo in this FINE-HEART analysis. When combined with the apparent lack of attenuation of cardiovascular and kidney benefits with finerenone among individuals with CKD but higher eGFR (*e.g*., ≥60 ml/min per 1.73 m^2^), these findings further support the introduction of finerenone at early stages of disease in eligible persons with CKD and type 2 diabetes to prevent/delay progression of CKD and related morbidity and mortality.^10^ The consistency of cardiovascular and kidney benefits of finerenone at lower levels of HbA_1c_ (*e.g*., <7%) additionally aligns with emerging nonglucocentric approaches to risk reduction in persons with type 2 diabetes.

When the broader population of individuals with type 2 diabetes and at least moderate-risk CKD (including >700 participants with UACR <30 mg/g) was evaluated in this analysis, benefits of finerenone on cardiovascular death or heart failure hospitalization appeared to be consistent across a broader range of UACR. While exploratory, this finding may support further study of finerenone in persons with CKD but UACR <30 mg/g, as even milder forms of albuminuria (*e.g*., ≥10–29 mg/g) have been associated with incremental risk of adverse cardiovascular outcomes, such as incident heart failure, stroke, myocardial infarction, and atrial fibrillation.^2^ However, in the primary analysis presented herein, treatment benefits of finerenone on the composite kidney outcome appeared greater at higher levels of UACR. Although strong evidence of heterogeneity for this outcome was not observed when a wider range of UACR was included, the limited number of events at lower levels of UACR underscores the need for cautious interpretation and further research. Evidence of attenuated kidney benefits among individuals with lower UACR in this analysis may also explain the observed trial-level heterogeneity for the composite kidney outcome in this analysis. Indeed, treatment benefits on kidney events in heart failure trials, which have generally enrolled persons at lower kidney risk, have been elusive.^26^ These findings suggest that it may be challenging to demonstrate short-term kidney benefits among individuals with lower UACR, and therein at low near-term risk of CKD progression.

In an exploratory analysis including FINEARTS-HF participants with CKD and at least moderate proteinuria but without diabetes, statistically significant benefits of finerenone on cardiovascular, kidney, and mortality outcomes were observed. Moreover, benefits of finerenone on cardiovascular and kidney outcomes in this larger population with a broader range of baseline glycemia appeared consistent irrespective of baseline HbA_1c_. These findings are consistent with and extend those of a prior analysis of FINE-HEART participants with type 2 diabetes^27^ and suggest that the cardiovascular and kidney benefits of finerenone may apply to individuals with CKD but without diabetes. However, given the relatively few individuals without diabetes included in this analysis (all of whom had heart failure), these findings should be confirmed in adequately powered clinical trials. Indeed, the effects of finerenone on eGFR slope are presently being evaluated in persons with albuminuric CKD but without diabetes in the FIND-CKD (NCT05047263) trial.^28^

The safety profile of finerenone in this analysis was consistent with prior trials.^11–13,17^ Risks of serious adverse events and hypokalemia were lower with finerenone compared with placebo, while risk of hyperkalemia was higher with finerenone compared with placebo. However, absolute risks of serious hyperkalemia events, such as serum potassium >6.0 mEq/L (4%), hyperkalemia-related treatment discontinuation (2%), and hyperkalemia-related hospitalization (1%), were low even in the context of high use of renin-angiotensin system inhibitors. These findings emphasize the favorable balance of benefit and risk with finerenone across a broad range of CKD and type 2 diabetes, especially when combined with adherence to guideline-based serum potassium monitoring strategies (*e.g*., 1 month after finerenone initiation and every 4 months thereafter).^8^

This analysis has some limitations. First, some end points, such as outpatient worsening heart failure events (part of the primary composite end point in FINEARTS-HF), could not be harmonized across the three trials. As such, this analysis may have underestimated the totality of treatment effects of finerenone on cardiovascular morbidity. Second, while exploratory analyses were performed to improve understanding of the effects of finerenone in broader CKD subpopulations, the numbers of FINE-HEART participants with lower UACR and without type 2 diabetes were low. As such, these analyses may have been underpowered, and further studies are needed to examine the effect of finerenone in these broader populations for which unmet need persists. Third, no adjustment for multiplicity was performed in this analysis. As such, there is potential for false discovery. Fourth, as the primary end point of cardiovascular death (exclusive of undetermined death) was narrowly missed in the overall FINE-HEART analysis,^17^ further subgroup analysis should be interpreted in this context. Fifth, additional research efforts are needed to ascertain whether these findings apply to younger individuals, to community settings, and individuals with more diverse racial and ethnic backgrounds.

Finerenone seems to improve morbidity and mortality across a wide spectrum of CKD in persons with type 2 diabetes. These findings additionally highlight the potential for clinically relevant benefits in broader populations with CKD, including those with lower levels of albuminuria or without diabetes, but further research efforts are needed.

## Supplementary Material

## Data Availability

Data belong to a third party, and authors are not authorized to share the data. Identity of Third Party: Bayer AG. Data are made available to qualified scientific and medical researchers through vivli.org. All requests will be reviewed by an independent scientific review panel and data provided according to the conditions reviewed at https://vivli.org/ourmember/Bayer/.

## Declarative Statements

This study includes clinical experimentation and received Institutional Review Board or Ethics Committee approval. All patients provided written informed consent. This study includes clinical experimentation and complies with the Declaration of Helsinki.

## Supplemental Material

Supplemental Table 1. Baseline characteristics of FINEARTS-HF participants excluded from the analysis.

Supplemental Table 2. Baseline characteristics of FINE-HEART participants with CKD and type 2 diabetes, by trial.

Supplemental Table 3. Treatment effects of finerenone versus placebo on morbidity and mortality in FINE-HEART participants with CKD and type 2 diabetes, after covariate adjustment.

Supplemental Table 4. Treatment effects of finerenone versus placebo on morbidity and mortality in FINE-HEART participants with CKD and type 2 diabetes, considering competing risk of all-cause death.

Supplemental Table 5. Treatment effects of finerenone versus placebo on morbidity and mortality in a sensitivity analysis including FIDELIO-DKD, FIGARO-DKD, and FINEARTS-HF participants with type 2 diabetes and at least moderate-risk CKD.

Supplemental Table 6. Treatment effects of finerenone versus placebo on morbidity and mortality in a sensitivity analysis including FIDELIO-DKD, FIGARO-DKD, and FINEARTS-HF participants with eGFR >25 ml/min per 1.73 m^2^ and UACR >30 mg/g, with or without type 2 diabetes.

Supplemental Table 7. Incidence of hyperkalemia and hypokalemia, according to baseline eGFR category.

Supplemental Figure 1. Distribution of kidney risk in FINE-HEART participants with CKD and type 2 diabetes.

Supplemental Figure 2. Treatment effects of finerenone versus placebo on the composite kidney outcome (eGFR ≥57%) among FINE-HEART participants with CKD and type 2 diabetes, according to baseline eGFR, UACR, and HbA_1c_.

Supplemental Figure 3. Effect of finerenone on the composite kidney outcome in FINE-HEART participants with CKD and type 2 diabetes, overall and in key subgroups.

Supplemental Figure 4. Treatment effects of finerenone versus placebo on cardiovascular and kidney outcomes in a sensitivity analysis including FIDELIO-DKD, FIGARO-DKD, and FINEARTS-HF participants with type 2 diabetes and at least moderate-risk CKD, according to baseline eGFR and UACR.

Supplemental Figure 5. Treatment effects of finerenone versus placebo on cardiovascular and kidney outcomes in a sensitivity analysis including FIDELIO-DKD, FIGARO-DKD, and FINEARTS-HF participants with eGFR >25 ml/min per 1.73 m^2^ and UACR >30 mg/g, with or without type 2 diabetes, according to baseline eGFR, UACR, and HbA_1c_.
