# Supplementary material for: Effect of Finerenone on Morbidity and Mortality in CKD
Source: J Am Soc Nephrol. 2025 Sep 12;37(2):312–25. doi: 10.1681/ASN.0000000823 (PMC12890010; doi:10.1681/ASN.0000000823)
Supplement: Supplementary file 1 [file jasn-37-312-s001.pdf]

ASN American Society of Nephrology
JASN Journal of the American Society of Nephrology
KIDNEY360 Accessing Our World From Every Angle
CJASN Clinical Journal of the American Society of Nephrology

ASN Journal Disclosure Form

As per ASN journal policy, I have disclosed any financial relationships or commitments I have held in the past 36 months as included below. I have listed my Current Employer below to indicate there is a relationship requiring disclosure. If no relationship exists, my Current Employer is not listed.

R. Agarwal reports the following:
Employer: Veteran's Administration (part time); Consultancy: Akebia, Bayer, Boehringer Ingelheim, Eli Lilly, Chinook, Alnylam, Vertex, Intercept Pharmaceuticals, Novartis; Honoraria: Akebia, Bayer, Boehringer Ingelheim, Chinook, Vertex, Intercept Pharmaceuticals, Novartis, Alnylam; Patents or Royalties: UpToDate; and Advisory or Leadership Role: Hypertension, NDT, Akebia, Bayer, Boehringer Ingelheim, Chinook, Vertex, Novartis, Alnylam.

I understand that the information above will be published within the journal article, if accepted, and that failure to comply and/or to accurately and completely report the potential financial conflicts of interest could lead to the following: 1) Prior to publication, article rejection, or 2) Post-publication, sanctions ranging from, but not limited to, issuing a correction, reporting the inaccurate information to the authors' institution, banning authors from submitting work to ASN journals for varying lengths of time, and/or retraction of the published work.

Name: Rajiv Agarwal
Manuscript ID: JASN-2025-000528R2
Manuscript Title: Effect of Finerenone on Morbidity and Mortality in CKD
Date of Completion: July 25, 2025
Disclosure Updated Date: July 25, 2025

ASN American Society of Nephrology | JASN Journal of the American Society of Nephrology | KIDNEY360 Accessing Our World From Every Angle | CJASN Clinical Journal of the American Society of Nephrology

ASN Journal Disclosure Form

As per ASN journal policy, I have disclosed any financial relationships or commitments I have held in the past 36 months as included below. I have listed my Current Employer below to indicate there is a relationship requiring disclosure. If no relationship exists, my Current Employer is not listed.

S. Anker reports the following:
Employer: Charité Medical School; Consultancy: Actimed, Alleviant, Astra Zeneca, Bayer, Berlin Heals, Boehringer Ingelheim, Brahms, Cardiac Dimensions, Cardior, Cordio, Corvia, CVRx, Cytokinetics, Edwards, Impulse Dynamics, Lilly, Mankind Pharma, Medtronic, Novo Nordisk, Occlutech, Pfizer, Regeneron, Relaxera, Repairon, Scirent, Sensible Medical, Vectorious, Vivus, and V-Wave.; Research Funding: Vifor Pharma, Abbott Vascular; Honoraria: Actimed, Alleviant, Astra Zeneca, Bayer, Berlin Heals, Boehringer Ingelheim, Brahms, Cardiac Dimensions, Cardior, Cordio, Corvia, CVRx, Cytokinetics, Edwards, Impulse Dynamics, Lilly, Mankind Pharma, Medtronic, Novo Nordisk, Occlutech, Pfizer, Regeneron, Relaxera, Repairon, Scirent, Sensible Medical, Vectorious, Vivus, and V-Wave.; Patents or Royalties: Named co-inventor of two patent applications regarding MR-proANP (DE 102007010834 & DE 102007022367), but he does not benefit personally from the related issued patents.; and Advisory or Leadership Role: Actimed, Alleviant, Astra Zeneca, Bayer, Berlin Heals, Boehringer Ingelheim, Brahms, Cardiac Dimensions, Cardior, Cordio, Corvia, CVRx, Cytokinetics, Edwards, Impulse Dynamics, Lilly, Mankind Pharma, Medtronic, Novo Nordisk, Occlutech, Pfizer, Regeneron, Relaxera, Repairon, Scirent, Sensible Medical, Vectorious, Vivus, and V-Wave.

I understand that the information above will be published within the journal article, if accepted, and that failure to comply and/or to accurately and completely report the potential financial conflicts of interest could lead to the following: 1) Prior to publication, article rejection, or 2) Post-publication, sanctions ranging from, but not limited to, issuing a correction, reporting the inaccurate information to the authors' institution, banning authors from submitting work to ASN journals for varying lengths of time, and/or retraction of the published work.

Name: Stefan D. Anker
Manuscript ID: JASN-2025-000528R3
Manuscript Title: Effect of Finerenone on Morbidity and Mortality in CKD
Date of Completion: August 14, 2025
Disclosure Updated Date: August 14, 2025

ASN American Society of Nephrology JASN Journal of the American Society of Nephrology KIDNEY360 Accessing Our World From Every Angle CJASN Clinical Journal of the American Society of Nephrology

ASN Journal Disclosure Form

As per ASN journal policy, I have disclosed any financial relationships or commitments I have held in the past 36 months as included below. I have listed my Current Employer below to indicate there is a relationship requiring disclosure. If no relationship exists, my Current Employer is not listed.

M. Brinker reports the following:
Employer: Bayer AG; Ownership Interest: Bayer AG; and Patents or Royalties: Description:; TREATMENT OF CHRONIC KIDNEY DISEASE IN TYPE I DIABETES MELLITUS; Patent Status:; Pending; Filing Jurisdiction:; EU; Patent Number:; WO2024/110523; Patent Holder:; Bayer.

I understand that the information above will be published within the journal article, if accepted, and that failure to comply and/or to accurately and completely report the potential financial conflicts of interest could lead to the following: 1) Prior to publication, article rejection, or 2) Post-publication, sanctions ranging from, but not limited to, issuing a correction, reporting the inaccurate information to the authors' institution, banning authors from submitting work to ASN journals for varying lengths of time, and/or retraction of the published work.

Name: Meike Daniela Brinker
Manuscript ID: JASN-2025-000528R2
Manuscript Title: Effect of Finerenone on Morbidity and Mortality in CKD
Date of Completion: July 25, 2025
Disclosure Updated Date: July 11, 2025

ASN
American Society of Nephrology
JASN
Journal of the American Society of Nephrology
KIDNEY360
Accessing Our World From Every Angle
CJASN
Clinical Journal of the American Society of Nephrology

ASN Journal Disclosure Form

As per ASN journal policy, I have disclosed any financial relationships or commitments I have held in the past 36 months as included below. I have listed my Current Employer below to indicate there is a relationship requiring disclosure. If no relationship exists, my Current Employer is not listed.

B. Claggett reports the following:
Employer: Harvard Medical School, Brigham and Women's Hospital; and Consultancy: Alnylam, Cardurion, Corvia, Cytokinetics, CVRx, Cardior, Eli Lilly, Intellia, Rocket.

I understand that the information above will be published within the journal article, if accepted, and that failure to comply and/or to accurately and completely report the potential financial conflicts of interest could lead to the following: 1) Prior to publication, article rejection, or 2) Post-publication, sanctions ranging from, but not limited to, issuing a correction, reporting the inaccurate information to the authors' institution, banning authors from submitting work to ASN journals for varying lengths of time, and/or retraction of the published work.

Name: Brian Claggett
Manuscript ID: JASN-2025-000528R1
Manuscript Title: Effect of Finerenone on Morbidity and Mortality in Chronic Kidney Disease
Date of Completion: July 10, 2025
Disclosure Updated Date: November 11, 2024

ASN
American Society of Nephrology
JASN
Journal of the American Society of Nephrology
KIDNEY360
Accessing Our World From Every Angle
CJASN
Clinical Journal of the American Society of Nephrology

ASN Journal Disclosure Form

As per ASN journal policy, I have disclosed any financial relationships or commitments I have held in the past 36 months as included below. I have listed my Current Employer below to indicate there is a relationship requiring disclosure. If no relationship exists, my Current Employer is not listed.

A. Desai reports the following:
Employer: Brigham and Women's Hospital; Consultancy: Abbott, /Alnylam, AstraZeneca, Avidity Biopharma, Axon Therapies, Bayer, Biofourmis, Boston Scientific, CVS, Endotronix, GlaxoSmithKline, Medpace, Medtronic, Merck, New Amsterdam, Novartis, Parexel, Regeneron, River2Renal, Roche, Veristat; Ownership Interest: Equity in DTx Plus; and Research Funding: Abbott, AstraZeneca, Alnylam, Bayer, Novartis, Pfizer.

I understand that the information above will be published within the journal article, if accepted, and that failure to comply and/or to accurately and completely report the potential financial conflicts of interest could lead to the following: 1) Prior to publication, article rejection, or 2) Post-publication, sanctions ranging from, but not limited to, issuing a correction, reporting the inaccurate information to the authors' institution, banning authors from submitting work to ASN journals for varying lengths of time, and/or retraction of the published work.

Name: Akshay Desai
Manuscript ID: JASN-2025-000528R3
Manuscript Title: Effect of Finerenone on Morbidity and Mortality in CKD
Date of Completion: August 10, 2025
Disclosure Updated Date: August 10, 2025

ASN American Society of Nephrology | JASN Journal of the American Society of Nephrology | KIDNEY360 Accessing Our World From Every Angle | CJASN Clinical Journal of the American Society of Nephrology

ASN Journal Disclosure Form

As per ASN journal policy, I have disclosed any financial relationships or commitments I have held in the past 36 months as included below. I have listed my Current Employer below to indicate there is a relationship requiring disclosure. If no relationship exists, my Current Employer is not listed.

G. Filippatos reports the following:
Employer: National and Kapodistrian University of Athens; Consultancy: Bayer; Medtronic; Novartis; Servier; Vifor; Boehringer Ingelheim, Impulse Dynamics, Cardior, Merck, Novo Nordisk;; Research Funding: No Pharma or device; European Union; Honoraria: Bayer, Boehringer Ingelheim, Cardior, Merck, Novo Nordisk; Advisory or Leadership Role: JACC HF; EJHF, President of Hellenic Heart failure Society; and Speakers Bureau: Boehringer Ingelheim, Bayer, Novo Nordisc.

I understand that the information above will be published within the journal article, if accepted, and that failure to comply and/or to accurately and completely report the potential financial conflicts of interest could lead to the following: 1) Prior to publication, article rejection, or 2) Post-publication, sanctions ranging from, but not limited to, issuing a correction, reporting the inaccurate information to the authors' institution, banning authors from submitting work to ASN journals for varying lengths of time, and/or retraction of the published work.

Name: Gerasimos Filippatos
Manuscript ID: JASN-2025-000528R1
Manuscript Title: "Effect of Finerenone on Morbidity and Mortality in Chronic Kidney Disease"
Date of Completion: July 4, 2025
Disclosure Updated Date: July 4, 2025

ASN
American Society of Nephrology
JASN
Journal of the American Society of Nephrology
KIDNEY360
Accessing Our World From Every Angle
CJASN
Clinical Journal of the American Society of Nephrology

ASN Journal Disclosure Form

As per ASN journal policy, I have disclosed any financial relationships or commitments I have held in the past 36 months as included below. I have listed my Current Employer below to indicate there is a relationship requiring disclosure. If no relationship exists, my Current Employer is not listed.

A. Henderson reports the following:
Employer: University of Glasgow; and Other Interests or Relationships: Bayer AG.

I understand that the information above will be published within the journal article, if accepted, and that failure to comply and/or to accurately and completely report the potential financial conflicts of interest could lead to the following: 1) Prior to publication, article rejection, or 2) Post-publication, sanctions ranging from, but not limited to, issuing a correction, reporting the inaccurate information to the authors' institution, banning authors from submitting work to ASN journals for varying lengths of time, and/or retraction of the published work.

Name: Alasdair D Henderson
Manuscript ID: JASN-2025-000528R1
Manuscript Title: Effect of Finerenone on Morbidity and Mortality in Chronic Kidney Disease
Date of Completion: July 11, 2025
Disclosure Updated Date: July 11, 2025

ASN American Society of Nephrology | JASN Journal of the American Society of Nephrology | KIDNEY360 Accessing Our World From Every Angle | CJASN Clinical Journal of the American Society of Nephrology

ASN Journal Disclosure Form

As per ASN journal policy, I have disclosed any financial relationships or commitments I have held in the past 36 months as included below. I have listed my Current Employer below to indicate there is a relationship requiring disclosure. If no relationship exists, my Current Employer is not listed.

P. Jhund reports the following:
Employer: University of Glasgow; Consultancy: AstraZeneca; Boehringer Ingelheim; Bayer AG; Ownership Interest: GCTP Ltd; Research Funding: Boheringer Ingelheim, Analog Devices, Astrazeneca, Roche Diagnostics,; Honoraria: AstraZeneca; Speakers Bureau: AstraZeneca; and Other Interests or Relationships: AstraZeneca, Bayer AG, Novo Nordisk has paid my employer the University of Glasgow for my time working on clinical trials.

I understand that the information above will be published within the journal article, if accepted, and that failure to comply and/or to accurately and completely report the potential financial conflicts of interest could lead to the following: 1) Prior to publication, article rejection, or 2) Post-publication, sanctions ranging from, but not limited to, issuing a correction, reporting the inaccurate information to the authors' institution, banning authors from submitting work to ASN journals for varying lengths of time, and/or retraction of the published work.

Name: Pardeep Jhund
Manuscript ID: ASN-2025-000528R1
Manuscript Title: Effect of Finerenone on Morbidity and Mortality in Chronic Kidney Disease
Date of Completion: July 2, 2025
Disclosure Updated Date: April 22, 2025

ASN American Society of Nephrology JASN Journal of the American Society of Nephrology KIDNEY360 Accessing Our World From Every Angle CJASN Clinical Journal of the American Society of Nephrology

ASN Journal Disclosure Form

As per ASN journal policy, I have disclosed any financial relationships or commitments I have held in the past 36 months as included below. I have listed my Current Employer below to indicate there is a relationship requiring disclosure. If no relationship exists, my Current Employer is not listed.

C. Lam reports the following:
Employer: National Heart Centre Singapore (Self); Us2.ai (Spouse); Consultancy: Alnylam Pharma, AnaCardio AB, Applied Therapeutics, AstraZeneca, Bayer, Biopeutics, Boehringer Ingelheim, Boston Scientific, Bristol Myers Squibb, Corteria, CPC Clinical Research, Cytokinetics, Eli Lilly, Impulse Dynamics, Intellia Therapeutics, Janssen Research & Development LLC, Medscape/WebMD Global LLC, Merck, Novartis, Novo Nordisk, Quidel Corporation, Radcliffe Group Ltd., Roche and Us2.ai; Ownership Interest: Us2.ai; Research Funding: NovoNordisk and Roche Diagnostics; Honoraria: Alnylam Pharma, AnaCardio AB, Applied Therapeutics, AstraZeneca, Bayer, Biopeutics, Boehringer Ingelheim, Boston Scientific, Bristol Myers Squibb, Corteria, CPC Clinical Research, Cytokinetics, Eli Lilly, Impulse Dynamics, Intellia Therapeutics, Ionis Pharmaceutical, Janssen Research & Development LLC, Medscape/WebMD Global LLC, Merck, Novartis, Novo Nordisk, Quidel Corporation, Radcliffe Group Ltd., Roche and Us2.ai; Patents or Royalties: 1. Patent pending: PCT/SG2016/050217; Method for diagnosis and prognosis of chronic heart failure; 2. US 10,631,828 B1 ; 3. US 10,702,247 B2 ; 4.. US 11,301,996 B2 ; 5. US 11,446,009 B2 ; 6. US 11,931,207 B2 ; 7. US 12,001,939;; and Advisory or Leadership Role: CEO of Us2.ai (Spouse); Non-executive director of Us2.ai (Self).

I understand that the information above will be published within the journal article, if accepted, and that failure to comply and/or to accurately and completely report the potential financial conflicts of interest could lead to the following: 1) Prior to publication, article rejection, or 2) Post-publication, sanctions ranging from, but not limited to, issuing a correction, reporting the inaccurate information to the authors' institution, banning authors from submitting work to ASN journals for varying lengths of time, and/or retraction of the published work.

Name: Carolyn S.P. Lam
Manuscript ID: JASN-2025-000528R3
Manuscript Title: Effect of Finerenone on Morbidity and Mortality in CKD
Date of Completion: August 6, 2025
Disclosure Updated Date: August 6, 2025

ASN American Society of Nephrology | JASN Journal of the American Society of Nephrology | KIDNEY360 Accessing Our World From Every Angle | CJASN Clinical Journal of the American Society of Nephrology

ASN Journal Disclosure Form

As per ASN journal policy, I have disclosed any financial relationships or commitments I have held in the past 36 months as included below. I have listed my Current Employer below to indicate there is a relationship requiring disclosure. If no relationship exists, my Current Employer is not listed.

J. McMurray reports the following:
Employer: University of Glasgow; Consultancy: Personal Consultancy fees: Alnylam Pharmaceuticals, AnaCardio, AstraZeneca, Bayer, Cardurion, Cytokinetics, Novartis, River BioMedics, Biohaven Pharmaceuticals, Chugai Pharmaceuticals, Protherics Medicine Developments Ltd., DalCor Pharmaceuticals.; Honoraria: Personal lecture fees: Alkem Metabolics, Astra Zeneca, Canadian Medical and Surgical Knowledge, Centrix Healthcare, Emcure Pharma, Eris Lifesciences, Hikma Pharma, Imagica Health, Intas Pharma, J.B. Chemicals & Pharma, Lupin Pharma, Medscape/Heart.Org., ProAdWise Communications, Radcliffe Cardiology, Sun Pharma, Translational Medicine Academy, Regeneron, MCI India, Hilton Pharma., IMEDIC Pharma Micro Labs Ltd., At the Limits Ltd., ARMGO Pharma.; Advisory or Leadership Role: Payments to Glasgow University for clinical trials and other research projects from the British Heart Foundation, National Institute for Health – National Heart Lung and Blood Institute (NIH-NHLBI), Alnylam Pharmaceuticals, AstraZeneca, Bayer, Cardurion, Cytokinetics, Novartis, Roche.; and Other Interests or Relationships: Data Safety Monitoring Boards: WCG Clinical Services. Director of Global Clinical Trial Partners Ltd (which provides clinical trial services such as endpoint committees and educational programmes).

I understand that the information above will be published within the journal article, if accepted, and that failure to comply and/or to accurately and completely report the potential financial conflicts of interest could lead to the following: 1) Prior to publication, article rejection, or 2) Post-publication, sanctions ranging from, but not limited to, issuing a correction, reporting the inaccurate information to the authors' institution, banning authors from submitting work to ASN journals for varying lengths of time, and/or retraction of the published work.

Name: John McMurray
Manuscript ID: JASN-2025-000528R1
Manuscript Title: Effect of Finerenone on Morbidity and Mortality in Chronic Kidney Disease
Date of Completion: July 3, 2025
Disclosure Updated Date: April 22, 2025

ASN American Society of Nephrology | JASN Journal of the American Society of Nephrology | KIDNEY360 Accessing Our World From Every Angle | CJASN Clinical Journal of the American Society of Nephrology

ASN Journal Disclosure Form

As per ASN journal policy, I have disclosed any financial relationships or commitments I have held in the past 36 months as included below. I have listed my Current Employer below to indicate there is a relationship requiring disclosure. If no relationship exists, my Current Employer is not listed.

Z. Miao reports the following:
Employer: Brigham and Women's Hospital

I understand that the information above will be published within the journal article, if accepted, and that failure to comply and/or to accurately and completely report the potential financial conflicts of interest could lead to the following: 1) Prior to publication, article rejection, or 2) Post-publication, sanctions ranging from, but not limited to, issuing a correction, reporting the inaccurate information to the authors' institution, banning authors from submitting work to ASN journals for varying lengths of time, and/or retraction of the published work.

Name: Zi Miao
Manuscript ID: JASN-2025-000528R1
Manuscript Title: Effect of Finerenone on Morbidity and Mortality in Chronic Kidney Disease
Date of Completion: July 14, 2025
Disclosure Updated Date: July 3, 2025

ASN American Society of Nephrology | JASN Journal of the American Society of Nephrology | KIDNEY360 Accessing Our World From Every Angle | CJASN Clinical Journal of the American Society of Nephrology

ASN Journal Disclosure Form

As per ASN journal policy, I have disclosed any financial relationships or commitments I have held in the past 36 months as included below. I have listed my Current Employer below to indicate there is a relationship requiring disclosure. If no relationship exists, my Current Employer is not listed.

J. Ostrominski reports the following:
Employer: Brigham and Women's Hospital; and Advisory or Leadership Role: Corcept Therapeutics.

I understand that the information above will be published within the journal article, if accepted, and that failure to comply and/or to accurately and completely report the potential financial conflicts of interest could lead to the following: 1) Prior to publication, article rejection, or 2) Post-publication, sanctions ranging from, but not limited to, issuing a correction, reporting the inaccurate information to the authors' institution, banning authors from submitting work to ASN journals for varying lengths of time, and/or retraction of the published work.

Name: John W. Ostrominski
Manuscript ID: JASN-2025-000528R3
Manuscript Title: Effect of Finerenone on Morbidity and Mortality in CKD
Date of Completion: August 1, 2025
Disclosure Updated Date: August 1, 2025

ASN American Society of Nephrology | JASN Journal of the American Society of Nephrology | KIDNEY360 Accessing Our World From Every Angle | CJASN Clinical Journal of the American Society of Nephrology

ASN Journal Disclosure Form

As per ASN journal policy, I have disclosed any financial relationships or commitments I have held in the past 36 months as included below. I have listed my Current Employer below to indicate there is a relationship requiring disclosure. If no relationship exists, my Current Employer is not listed.

There is no minimum financial threshold; individuals must disclose all financial relationships, regardless of the amount, with ineligible companies. Individuals must disclose for every category below, regardless of their view of the relevance of the relationship to the activity. ("*Ineligible companies" are those whose primary business is producing, marketing, selling, re-selling, or distributing health care products used by or on patients.*)

Date: 05 September 2025

Author Name: Bertram Pitt, MD

Manuscript ID: JASN-2025-000528R3

Manuscript Title: Effect of Finerenone on Morbidity and Mortality in CKD

Disclosure Statement: *(including all categories below*)*

Dr. Pitt has served as a consultant for Bayer, Boehringer Ingelheim, Lexicon, Astra Zeneca, Vifor, ScPharmaceuticals, Sqinnovations, G3 Pharmaceuticals, Sarfez Phaemaceuticals, KBP Biosciences, Cereno Scientific, Anacardio, Prointel, and Sea Star Medical. He has stock/stock options with Vifor, ScPharmaceuticals, Sqinnovations, Sarfez Phaemaceuticals, KBP Biosciences, Cereno Scientific, Anacardio, Prointel, and Sea Star Medical. He serves on a DSMB for Mineralis.
He holds the following US Patents:
US Patent 9931412 site specific delivery of eplerenone to the myocardium
US Patent pending 63/045,783 Histone modulating agents for the prevention and treatment of organ damage

***Categories:** Employer; Ownership Interest; Consultancy; Research Funding; Honoraria; Patents or Royalties; Advisory or Leadership Role; Speakers Bureau; and Other Interests or Relationships

Author Acknowledgment: I understand that the information above will be published within the journal article, if accepted, and that failure to comply and/or to accurately and completely report the potential financial conflicts of interest could lead to the following: 1) Prior to publication, article rejection, or 2) Post-publication, sanctions ranging from, but not limited to, issuing a correction, reporting the inaccurate information to the authors' institution, banning authors from submitting work to ASN journals for varying lengths of time, and/or retraction of the published work.

Author Name and/or Initials: Bertram Pitt

ASN American Society of Nephrology | JASN Journal of the American Society of Nephrology | KIDNEY360 Accessing Our World From Every Angle | CJASN Clinical Journal of the American Society of Nephrology

ASN Journal Disclosure Form

As per ASN journal policy, I have disclosed any financial relationships or commitments I have held in the past 36 months as included below. I have listed my Current Employer below to indicate there is a relationship requiring disclosure. If no relationship exists, my Current Employer is not listed.

K. Rohwedder reports the following:
Employer: Bayer AG

I understand that the information above will be published within the journal article, if accepted, and that failure to comply and/or to accurately and completely report the potential financial conflicts of interest could lead to the following: 1) Prior to publication, article rejection, or 2) Post-publication, sanctions ranging from, but not limited to, issuing a correction, reporting the inaccurate information to the authors' institution, banning authors from submitting work to ASN journals for varying lengths of time, and/or retraction of the published work.

Name: Katja Rohwedder
Manuscript ID: JASN-2025-000528R1
Manuscript Title: Effect of Finerenone on Morbidity and Mortality in Chronic Kidney Disease
Date of Completion: July 7, 2025
Disclosure Updated Date: July 3, 2025

ASN American Society of Nephrology JASN Journal of the American Society of Nephrology KIDNEY360 Accessing Our World From Every Angle CJASN Clinical Journal of the American Society of Nephrology

ASN Journal Disclosure Form

As per ASN journal policy, I have disclosed any financial relationships or commitments I have held in the past 36 months as included below. I have listed my Current Employer below to indicate there is a relationship requiring disclosure. If no relationship exists, my Current Employer is not listed.

P. Rossing reports the following:
Employer: Steno Diabetes Center Copenhagen; Research Funding: Novo Nordisk , AstraZeneca, Bayer, Lexicon Pharma; Honoraria: Boehringer Ingelheim, AstraZeneca, Abbott, Novo Nordisk, all honoraria to institution; and Advisory or Leadership Role: Astra Zeneca Bayer , Novo Nordisk, Gilead all honoraria to institution.

I understand that the information above will be published within the journal article, if accepted, and that failure to comply and/or to accurately and completely report the potential financial conflicts of interest could lead to the following: 1) Prior to publication, article rejection, or 2) Post-publication, sanctions ranging from, but not limited to, issuing a correction, reporting the inaccurate information to the authors' institution, banning authors from submitting work to ASN journals for varying lengths of time, and/or retraction of the published work.

Name: Peter Rossing
Manuscript ID: JASN-2025-000528R1
Manuscript Title: ("Effect of Finerenone on Morbidity and Mortality in Chronic Kidney Disease
Date of Completion: July 3, 2025
Disclosure Updated Date: March 19, 2025

ASN Journal Disclosure Form

As per ASN journal policy, I have disclosed any financial relationships or commitments I have held in the past 36 months as included below. I have listed my Current Employer below to indicate there is a relationship requiring disclosure. If no relationship exists, my Current Employer is not listed.

L. Ruilope reports the following:
Employer: National Institute of Health, Spain; Universidad Europea de Madrid, Spain; and Honoraria: As speaker for Bayer AG.

I understand that the information above will be published within the journal article, if accepted, and that failure to comply and/or to accurately and completely report the potential financial conflicts of interest could lead to the following: 1) Prior to publication, article rejection, or 2) Post-publication, sanctions ranging from, but not limited to, issuing a correction, reporting the inaccurate information to the authors' institution, banning authors from submitting work to ASN journals for varying lengths of time, and/or retraction of the published work.

Name: Luis M. Ruilope
Manuscript ID: JASN-2025-000528R2
Manuscript Title: Effect of Finerenone on Morbidity and Mortality in CKD
Date of Completion: August 10, 2025
Disclosure Updated Date: August 10, 2025

ASN American Society of Nephrology | JASN Journal of the American Society of Nephrology | KIDNEY360 Accessing Our World From Every Angle | CJASN Clinical Journal of the American Society of Nephrology

ASN Journal Disclosure Form

As per ASN journal policy, I have disclosed any financial relationships or commitments I have held in the past 36 months as included below. I have listed my Current Employer below to indicate there is a relationship requiring disclosure. If no relationship exists, my Current Employer is not listed.

A. Scalise reports the following:
Employer: Bayer Hispania S.L

I understand that the information above will be published within the journal article, if accepted, and that failure to comply and/or to accurately and completely report the potential financial conflicts of interest could lead to the following: 1) Prior to publication, article rejection, or 2) Post-publication, sanctions ranging from, but not limited to, issuing a correction, reporting the inaccurate information to the authors' institution, banning authors from submitting work to ASN journals for varying lengths of time, and/or retraction of the published work.

Name: Andrea Scalise
Manuscript ID: JASN-2025-000528R2
Manuscript Title: Effect of Finerenone on Morbidity and Mortality in CKD
Date of Completion: July 25, 2025
Disclosure Updated Date: February 19, 2025

ASN
American Society of Nephrology
JASN
Journal of the American Society of Nephrology
KIDNEY360
Accessing Our World From Every Angle
CJASN
Clinical Journal of the American Society of Nephrology

ASN Journal Disclosure Form

As per ASN journal policy, I have disclosed any financial relationships or commitments I have held in the past 36 months as included below. I have listed my Current Employer below to indicate there is a relationship requiring disclosure. If no relationship exists, my Current Employer is not listed.

P. Schloemer reports the following:
Employer: Bayer AG

I understand that the information above will be published within the journal article, if accepted, and that failure to comply and/or to accurately and completely report the potential financial conflicts of interest could lead to the following: 1) Prior to publication, article rejection, or 2) Post-publication, sanctions ranging from, but not limited to, issuing a correction, reporting the inaccurate information to the authors' institution, banning authors from submitting work to ASN journals for varying lengths of time, and/or retraction of the published work.

Name: Patrick Schloemer
Manuscript ID: JASN-2025-000528R3
Manuscript Title: Effect of Finerenone on Morbidity and Mortality in CKD
Date of Completion: August 6, 2025
Disclosure Updated Date: May 23, 2025

ASN Journal Disclosure Form

As per ASN journal policy, I have disclosed any financial relationships or commitments I have held in the past 36 months as included below. I have listed my Current Employer below to indicate there is a relationship requiring disclosure. If no relationship exists, my Current Employer is not listed.

M. Senni reports the following:
Employer: UNIVERSITY BICOCCA MILAN ITALY; Consultancy: Novartis, Bayer, Merck, MSD, Boehringer, Abbott, Astrazeneca, Vifor , Novonordisk, Cardurion, Amgen; Honoraria: Novartis, Bayer, Merck, MSD, Boehringer, Abbott, Astrazeneca, Vifor , Novonordisk, Cardurion, Amgen; Advisory or Leadership Role: Novartis, Bayer, Merck, MSD, Boehringer, Abbott, Astrazeneca, Vifor , Novonordisk, Cardurion, Amgen; and Speakers Bureau: Novartis, Bayer, Merck, MSD, Boehringer, Abbott, Astrazeneca, Vifor , Novonordisk, Cardurion, Amgen.

I understand that the information above will be published within the journal article, if accepted, and that failure to comply and/or to accurately and completely report the potential financial conflicts of interest could lead to the following: 1) Prior to publication, article rejection, or 2) Post-publication, sanctions ranging from, but not limited to, issuing a correction, reporting the inaccurate information to the authors' institution, banning authors from submitting work to ASN journals for varying lengths of time, and/or retraction of the published work.

Name: Michele Senni
Manuscript ID: b3abfedb669d280b
Manuscript Title: Effect of Finerenone on Morbidity and Mortality in Chronic Kidney Disease,
Date of Completion: July 3, 2025
Disclosure Updated Date: July 3, 2025

ASN American Society of Nephrology
JASN Journal of the American Society of Nephrology
KIDNEY360 Accessing Our World From Every Angle
CJASN Clinical Journal of the American Society of Nephrology

ASN Journal Disclosure Form

As per ASN journal policy, I have disclosed any financial relationships or commitments I have held in the past 36 months as included below. I have listed my Current Employer below to indicate there is a relationship requiring disclosure. If no relationship exists, my Current Employer is not listed.

S. Shah reports the following:
Employer: Northwestern University; and Consultancy: Bayer.

I understand that the information above will be published within the journal article, if accepted, and that failure to comply and/or to accurately and completely report the potential financial conflicts of interest could lead to the following: 1) Prior to publication, article rejection, or 2) Post-publication, sanctions ranging from, but not limited to, issuing a correction, reporting the inaccurate information to the authors' institution, banning authors from submitting work to ASN journals for varying lengths of time, and/or retraction of the published work.

Name: Sanjiv Shah
Manuscript ID: JASN-2025-000528R1
Manuscript Title: Effect of Finerenone on Morbidity and Mortality in Chronic Kidney Disease
Date of Completion: July 11, 2025
Disclosure Updated Date: April 21, 2025

ASN American Society of Nephrology | JASN Journal of the American Society of Nephrology | KIDNEY360 Accessing Our World From Every Angle | CJASN Clinical Journal of the American Society of Nephrology

ASN Journal Disclosure Form

As per ASN journal policy, I have disclosed any financial relationships or commitments I have held in the past 36 months as included below. I have listed my Current Employer below to indicate there is a relationship requiring disclosure. If no relationship exists, my Current Employer is not listed.

S. Solomon reports the following:
Employer: Brigham and Women's Hospital; Consultancy: Abbott, Action, Akros, Alnylam, Amgen, Arena, AstraZeneca, Bayer, Boeringer-Ingelheim, BMS, Cardior, Cardurion, Corvia, Cytokinetics, Daiichi-Sankyo, GSK, Lilly, Merck, Myokardia, Novartis, Roche, Theracos, Quantum Genomics, Cardurion, Janssen, Cardiac Dimensions, Tenaya, Sanofi-Pasteur, Dinaqor, Tremeau, CellProThera, Moderna, American Regent, Sarepta, Lexicon, Anacardio, Akros, Valo; and Research Funding: Actelion, Alnylam, Amgen, AstraZeneca, Bellerophon, Bayer, BMS, Celladon, Cytokinetics, Eidos, Gilead, GSK, Ionis, Lilly, Mesoblast, MyoKardia, NIH/NHLBI, Neurotronik, Novartis, NovoNordisk, Respicardia, Sanofi Pasteur, Theracos, US2.AI, Edgewise.

I understand that the information above will be published within the journal article, if accepted, and that failure to comply and/or to accurately and completely report the potential financial conflicts of interest could lead to the following: 1) Prior to publication, article rejection, or 2) Post-publication, sanctions ranging from, but not limited to, issuing a correction, reporting the inaccurate information to the authors' institution, banning authors from submitting work to ASN journals for varying lengths of time, and/or retraction of the published work.

Name: Scott D. Solomon
Manuscript ID: JASN-2025-000528R3
Manuscript Title: Effect of Finerenone on Morbidity and Mortality in CKD
Date of Completion: August 5, 2025
Disclosure Updated Date: January 23, 2025

ASN
American Society of Nephrology
JASN
Journal of the American Society of Nephrology
KIDNEY360
Accessing Our World From Every Angle
CJASN
Clinical Journal of the American Society of Nephrology

ASN Journal Disclosure Form

As per ASN journal policy, I have disclosed any financial relationships or commitments I have held in the past 36 months as included below. I have listed my Current Employer below to indicate there is a relationship requiring disclosure. If no relationship exists, my Current Employer is not listed.

M. Vaduganathan reports the following:
Employer: Brigham and Women's Hospital, Harvard Medical School; Consultancy: Alnylam Pharmaceuticals, American Regent, Amgen, AstraZeneca, Bayer AG, Baxter Healthcare, BMS, Boehringer Ingelheim, Chiesi, Cytokinetics, Esperion, Fresenius Medical Care, Idorsia Pharmaceuticals, Lexicon Pharmaceuticals, Merck, Milestone Pharmaceuticals, Novartis, Novo Nordisk, Pharmacosmos, Recordati, Relypsa, Roche Diagnostics, Sanofi, and Tricog Health; Research Funding: Amgen, AstraZeneca, Boehringer Ingelheim, Galmed, Novartis, Bayer AG, Occlutech, Pharmacosmos, and Impulse Dynamics.; and Speakers Bureau: AstraZeneca, Boehringer Ingelheim, Novartis, Roche Diagnostics, Lexicon Pharmaceuticals, Cytokinetics.

I understand that the information above will be published within the journal article, if accepted, and that failure to comply and/or to accurately and completely report the potential financial conflicts of interest could lead to the following: 1) Prior to publication, article rejection, or 2) Post-publication, sanctions ranging from, but not limited to, issuing a correction, reporting the inaccurate information to the authors' institution, banning authors from submitting work to ASN journals for varying lengths of time, and/or retraction of the published work.

Name: Muthiah Vaduganathan
Manuscript ID: CJASN-2025-000711R2
Manuscript Title: Effect of finerenone on eGFR slope across different levels of baseline albuminuria and eGFR: Insights from FINEARTS-HF
Date of Completion: August 4, 2025
Disclosure Updated Date: August 4, 2025

ASN
American Society of Nephrology
JASN
Journal of the American Society of Nephrology
KIDNEY360
Accessing Our World From Every Angle
CJASN
Clinical Journal of the American Society of Nephrology

ASN Journal Disclosure Form

As per ASN journal policy, I have disclosed any financial relationships or commitments I have held in the past 36 months as included below. I have listed my Current Employer below to indicate there is a relationship requiring disclosure. If no relationship exists, my Current Employer is not listed.

A. Voors reports the following:
Employer: UMCG; Consultancy: The employer of AAV received consultancy fees and/or research support from Adrenomed, Anacardio, AstraZeneca, Bayer AG, BMS, Boehringer Ingelheim, Cardurion, Corteria, EliLilly, Merck, Moderna, Novartis, Novo Nordisk, Rycarma , SalubrisBio.; Ownership Interest: UMCG; and Research Funding: The employer of AAV received research support from Adrenomed, Bayer AG, BMS, Boehringer Ingelheim, Corteria, Merck, Novartis, Novo Nordisk, Roche diagnostics, SalubrisBio.

I understand that the information above will be published within the journal article, if accepted, and that failure to comply and/or to accurately and completely report the potential financial conflicts of interest could lead to the following: 1) Prior to publication, article rejection, or 2) Post-publication, sanctions ranging from, but not limited to, issuing a correction, reporting the inaccurate information to the authors' institution, banning authors from submitting work to ASN journals for varying lengths of time, and/or retraction of the published work.

Name: Adriaan A. Voors
Manuscript ID: JASN-2025-000528R1
Manuscript Title: Effect of Finerenone on Morbidity and Mortality in Chronic Kidney Disease
Date of Completion: July 3, 2025
Disclosure Updated Date: July 3, 2025

ASN American Society of Nephrology | JASN Journal of the American Society of Nephrology | KIDNEY360 Accessing Our World From Every Angle | CJASN Clinical Journal of the American Society of Nephrology

ASN Journal Disclosure Form

As per ASN journal policy, I have disclosed any financial relationships or commitments I have held in the past 36 months as included below. I have listed my Current Employer below to indicate there is a relationship requiring disclosure. If no relationship exists, my Current Employer is not listed.

F. Zannad reports the following:
Employer: Inserm, CHU & Université de Lorraine; Consultancy: Alnylam, Bayer, Biopeutics, Boehringer, Cellprothera, Cereno, Centrix, Corteria, CVRx, CVCT, Lilly, Lupin, Merck, NovoNordisk, Opalia Recordati, Owkin, Ribocure, Roche, Viatris.; Ownership Interest: Cereno, CVCT; Honoraria: Alnylam, Bayer, Biopeutics, Boehringer, Cellprothera, Cereno, Centrix, Corteria, CVRx, CVCT, Lilly, Lupin, Merck, NovoNordisk, Opalia Recordati, Owkin, Ribocure, Riche, Viatris.; Advisory or Leadership Role: Alnylam, Bayer, Biopeutics, Boehringer, Cellprothera, Cereno, Corteria, CVRx, Merck, Owkin, Ribocure, Roche; Speakers Bureau: Bayer, Boehringer, Centrix, CVRx, Lupin, Opalia Recordati, Merck, NovoNordisk, Viatris.; and Other Interests or Relationships: Polygon, Cereno pharmaceutical and CVCT.

I understand that the information above will be published within the journal article, if accepted, and that failure to comply and/or to accurately and completely report the potential financial conflicts of interest could lead to the following: 1) Prior to publication, article rejection, or 2) Post-publication, sanctions ranging from, but not limited to, issuing a correction, reporting the inaccurate information to the authors' institution, banning authors from submitting work to ASN journals for varying lengths of time, and/or retraction of the published work.

Name: Faiez Zannad
Manuscript ID: JASN-2025-000528R1
Manuscript Title: Effect of Finerenone on Morbidity and Mortality in Chronic Kidney Disease,
Date of Completion: July 5, 2025
Disclosure Updated Date: July 5, 2025
