# Supplementary material for: Effect of Finerenone on Morbidity and Mortality in CKD
Source: J Am Soc Nephrol. 2025 Sep 12;37(2):312–25. doi: 10.1681/ASN.0000000823 (PMC12890010; doi:10.1681/ASN.0000000823)
Supplement: Supplementary file 2 [file jasn-37-312-s002.pdf]

Effect of Finerenone on Morbidity and Mortality in CKD

Supplemental Material

Table of Contents

- Tables
  - Supplemental Table 1: Baseline Characteristics of FINEARTS-HF Participants Excluded from the Analysis
  - Supplemental Table 2: Baseline Characteristics of FINE-HEART Participants with Chronic Kidney Disease and Type 2 Diabetes, by Trial
  - Supplemental Table 3: Treatment Effects of Finerenone vs. Placebo on Morbidity and Mortality in FINE-HEART Participants with Chronic Kidney Disease and Type 2 Diabetes, After Covariate Adjustment
  - Supplemental Table 4: Treatment Effects of Finerenone vs. Placebo on Morbidity and Mortality in FINE-HEART Participants with Chronic Kidney Disease and Type 2 Diabetes, Considering Competing Risk of All-Cause Death
  - Supplemental Table 5: Treatment Effects of Finerenone vs. Placebo on Morbidity and Mortality in a Sensitivity Analysis Including FIDELIO-DKD, FIGARO-DKD, and FINEARTS-HF Participants with Type 2 Diabetes and At Least Moderate-Risk Chronic Kidney Disease
  - Supplemental Table 6: Treatment Effects of Finerenone vs. Placebo on Morbidity and Mortality in a Sensitivity Analysis Including FIDELIO-DKD, FIGARO-DKD, and FINEARTS-HF Participants with eGFR >25 mL/min/1.73 m^2 and UACR >30 mg/g, With or Without Type 2 Diabetes
  - Supplemental Table 7: Incidence of Hyperkalemia and Hypokalemia, According to Baseline eGFR Category

- Figures
  - Supplemental Figure 1: Distribution of Kidney Risk in FINE-HEART Participants with Chronic Kidney Disease and Type 2 Diabetes
  - Supplemental Figure 2: Treatment Effects of Finerenone vs. Placebo on the Composite Kidney Outcome (eGFR ≥57%) Among FINE-HEART Participants with Chronic Kidney Disease and Type 2 Diabetes, According to Baseline eGFR, UACR, and HbA_{1c}
  - Supplemental Figure 3: Effect of Finerenone on the Composite Kidney Outcome in FINE-HEART Participants with Chronic Kidney Disease and Type 2 Diabetes, Overall and in Key Subgroups
  - Supplemental Figure 4: Treatment Effects of Finerenone vs. Placebo on Cardiovascular and Kidney Outcomes in a Sensitivity Analysis Including FIDELIO-DKD, FIGARO-DKD, and FINEARTS-HF Participants with Type 2 Diabetes and At Least Moderate-Risk Chronic Kidney Disease, According to Baseline eGFR and UACR
  - Supplemental Figure 5: Treatment Effects of Finerenone vs. Placebo on Cardiovascular and Kidney Outcomes in a Sensitivity Analysis Including FIDELIO-DKD, FIGARO-DKD, and FINEARTS-HF Participants with eGFR >25 mL/min/1.73 m^2 and UACR >30 mg/g, With or Without Type 2 Diabetes, According to Baseline eGFR, UACR, and HbA_{1c}

Supplemental Table 1: Baseline Characteristics of FINEARTS-HF Participants Excluded from the Analysis

Characteristic	Included in Main Analysis (n=14,180)	Excluded from Main Analysis (n=4,811)
Age, y	65 ± 10	72 ± 10
Female	4417 (31%)	2247 (47%)
**Race**[a]		
Asian	3086 (22%)	770 (16%)
Black	535 (4%)	73 (2%)
Other	773 (6%)	150 (3%)
White	9786 (69%)	3818 (79%)
**Region**		
Asia	2862 (20%)	761 (16%)
Eastern Europe	3738 (26%)	2204 (46%)
Latin America	1579 (11%)	496 (10%)
North America	2167 (15%)	353 (7%)
Western Europe, Oceania and Others	3834 (27%)	997 (21%)
Body mass index, kg/m^2	31 ± 6	30 ± 6
Waist circumference, cm	107 ± 15	103 ± 16
Waist-hip ratio	1.0 ± 0.1	1.0 ± 0.1
Systolic blood pressure, mm Hg	136 ± 14	129 ± 15
Potassium, mEq/L	4.4 ± 0.4	4.4 ± 0.5
HbA_{1c}, %	7.7 ± 1.4	6.2 ± 1.0
eGFR, mL/min/1.73 m^2	58 ± 22	63 ± 20
**eGFR category, mL/min/1.73 m^2**		
<25	162 (1%)	32 (1%)
25 to <45	4605 (33%)	919 (19%)
45 to <60	3762 (27%)	1220 (25%)
≥60	5648 (40%)	2640 (55%)
History of heart failure	2197 (16%)	4811 (100%)
History of atrial fibrillation	1718 (12%)	2659 (55%)
Atrial fibrillation on electrocardiogram	984 (7%)	1844 (38%)
Duration of diabetes, years	15 ± 9	10 ± 9
**Duration of diabetes category, years**		
<5	1612 (11%)	433 (35%)
≥5 to <10	2538 (18%)	268 (22%)
≥10 to <15	3058 (22%)	233 (19%)
≥15 to <20	2974 (21%)	139 (11%)
≥20 to <25	2026 (14%)	94 (8%)
≥25	1941 (14%)	70 (6%)
**Background medication use**		
Diuretics	7875 (56%)	4756 (99%)
ACEi	5447 (38%)	1784 (37%)
ARB	8357 (59%)	1647 (34%)
Aspirin	6801 (48%)	1515 (32%)
Statin	10279 (73%)	3158 (66%)
SGLT2i	1194 (8%)	496 (10%)
GLP-1RA	1045 (7%)	65 (1%)
Potassium lowering therapies[b]	189 (1%)	6 (0.1%)

Values reported as n (%) or mean ± SD.
[a]Represents self-reported race. Participants electing to not disclose race or who self-identified as multiple races are included in the "Other" category for descriptive purposes.
[b]Includes patiromer, sodium polystyrene sulfonate, calcium polystyrene sulfonate
Abbreviations: ACEi = angiotensin converting enzyme inhibitors; ARB = angiotensin receptor blocker; eGFR = estimated glomerular filtration rate; GLP-1RA = glucagon-like peptide-1 receptor agonist; HbA_{1c} = glycated hemoglobin; KDIGO = Kidney Disease: Improving Global Outcomes; SGLT2i = sodium-glucose co-transporter 2 inhibitor; UACR = urine albumin-to-creatinine ratio

Supplemental Table 2: Baseline Characteristics of FINE-HEART Participants with Chronic Kidney Disease and Type 2 Diabetes, by Trial

Characteristic	FIDELIO-DKD and FIGARO-DKD (n=12990)	FINEARTS-HF (n=1190)
Age, y	65 ± 10	72 ± 9
Female	3932 (30%)	485 (41%)
**Race[a]**		
Asian	2860 (22%)	226 (19%)
Black	520 (4%)	15 (1%)
Other	741 (6%)	32 (3%)
White	8869 (68%)	917 (77%)
**Region**		
Asia	2640 (20%)	222 (19%)
Eastern Europe	3292 (25%)	446 (38%)
Latin America	1434 (11%)	145 (12%)
North America	2049 (16%)	118 (10%)
Western Europe, Oceania and Others	3575 (28%)	259 (22%)
Body mass index, kg/m^2	31 ± 6	31 ± 6
Waist circumference, cm	107 ± 15	107 ± 16
Waist-hip ratio	1.0 ± 0.1	1.0 ± 0.1
Systolic blood pressure, mm Hg	137 ± 14	133 ± 16
Potassium, mEq/L	4.3 ± 0.4	4.4 ± 0.5
HbA_{1c}, %	7.7 ± 1.4	7.4 ± 1.4
eGFR, mL/min/1.73 m^2	58 ± 22	57 ± 20
**eGFR category, mL/min/1.73 m^2**		
<25	162 (1%)	0 (0%)
25 to <45	4224 (33%)	381 (32%)
45 to <60	3426 (26%)	336 (28%)
≥60	5175 (40%)	473 (40%)
UACR, mg/g	515 [198, 1148]	132 [55, 435]
**Baseline UACR category, mg/g**		
<30	230 (2%)	0 (0%)
30 to <300	4081 (31%)	806 (68%)
≥300	8674 (67%)	384 (32%)
**KDIGO risk category**		
Low	64 (1%)	0 (0%)
Moderately increased	1313 (10.%)	349 (29%)
High	5335 (41%)	350 (29%)
Very high	6278 (48%)	491 (41%)
History of heart failure	1007 (8%)	1190 (100%)
History of atrial fibrillation	1104 (9%)	614 (52%)
Atrial fibrillation on electrocardiogram	535 (4%)	449 (38%)
Duration of diabetes, years	15 ± 9	12 ± 9
**Duration of diabetes category, years**		
<5	1306 (10%)	306 (26%)
≥5 to <10	2284 (18%)	254 (22%)
≥10 to <15	2820 (22%)	238 (20%)
≥15 to <20	2832 (22%)	142 (12%)
≥20 to <25	1905 (15%)	121 (10%)
≥25	1825 (14%)	116 (10%)
**Background medication use**		
Diuretics	6701 (52%)	1174 (99%)
ACEi	5076 (39%)	371 (31%)
ARB	7902 (61%)	455 (38%)
Aspirin	6368 (49%)	433 (36%)
Statin	9387 (72%)	892 (75%)
SGLT2i	873 (7%)	321 (27%)
GLP-1RA	943 (7%)	102 (9%)
Potassium lowering therapies[b]	182 (1%)	7 (1%)

Values reported as n (%), mean ± SD, or median [IQR].
[a]Represents self-reported race. Participants electing to not disclose race or who self-identified as multiple races are included in the "Other" category for descriptive purposes.
[b]Includes patiromer, sodium polystyrene sulfonate, calcium polystyrene sulfonate
Abbreviations: ACEi = angiotensin converting enzyme inhibitors; ARB = angiotensin receptor blocker; eGFR = estimated glomerular filtration rate; GLP-1RA = glucagon-like peptide-1 receptor agonist; HbA_{1c} = glycated hemoglobin; KDIGO = Kidney Disease: Improving Global Outcomes; SGLT2i = sodium-glucose co-transporter 2 inhibitor; UACR = urine albumin-to-creatinine ratio

Supplemental Table 3: Treatment Effects of Finerenone vs. Placebo on Morbidity and Mortality in FINE-HEART Participants with Chronic Kidney Disease and Type 2 Diabetes, After Covariate Adjustment

Outcomes	Finerenone vs. Placebo aHR[a] (95% CI)
**Cardiovascular events[b]**	
Cardiovascular death or heart failure hospitalization	0.82 (0.74, 0.92)
Cardiovascular death	0.84 (0.70, 0.99)
Heart failure hospitalization	0.80 (0.70, 0.92)
Major adverse cardiovascular events	0.89 (0.82, 0.98)
New-onset atrial fibrillation	0.84 (0.71, 1.00)
**Cardiovascular events[c]**	
Cardiovascular death or heart failure hospitalization	0.85 (0.77, 0.94)
Cardiovascular death	0.87 (0.76, 1.00)
Major adverse cardiovascular events	0.89 (0.82, 0.97)
**Kidney outcomes**	
Composite kidney outcome (eGFR ≥50%)[d]	0.78 (0.69, 0.88)
Composite kidney outcome (eGFR ≥57%)[e]	0.77 (0.67, 0.89)
**All-cause morbidity and mortality**	
All-cause death	0.91 (0.82, 1.01)
All-cause hospitalization	0.96 (0.92, 1.01)
All-cause death or all-cause hospitalization	0.96 (0.91, 1.00)

[a]: Estimated from Cox proportional hazards regression model inclusive of age, sex, race, baseline body mass index, baseline systolic blood pressure, baseline diabetes duration, baseline glycated hemoglobin, baseline eGFR, baseline potassium level, history of heart failure, history of atrial fibrillation, presence of atrial fibrillation on baseline electrocardiogram, angiotensin-converting enzyme inhibitor use, angiotensin receptor blocker use, angiotensin receptor-neprilysin inhibitor use, diuretic use, sodium-glucose co-transporter 2 inhibitor use, glucagon-like peptide-1 receptor agonist use, potassium-lowering medication use, aspirin use, statin use, and log-transformed baseline urine albumin-to-creatinine ratio. Models additionally stratified by trial and geographic region.
[b]: Endpoints inclusive of cardiovascular death exclusive of deaths with undetermined causes.
[c]: Endpoints inclusive of cardiovascular death inclusive of deaths with undetermined causes.
[d]: Reflects time to first sustained decrease in estimated glomerular filtration rate to ≥50% from baseline, sustained decrease in estimated glomerular filtration rate to <15 mL/min/1.73 m^2, kidney failure and death due to kidney failure.
[e]: Reflects time to first sustained decrease in estimated glomerular filtration rate to ≥57% from baseline, sustained decrease in estimated glomerular filtration rate to <15 mL/min/1.73 m^2, kidney failure and death due to kidney failure.
Major adverse cardiovascular events reflects the composite of non-fatal myocardial infarction, non-fatal stroke, heart failure hospitalization, and cardiovascular death.
Abbreviations: eGFR = estimated glomerular filtration rate; HR = hazard ratio; py = person-years

Supplemental Table 4: Treatment Effects of Finerenone vs. Placebo on Morbidity and Mortality in FINE-HEART Participants with Chronic Kidney Disease and Type 2 Diabetes, Considering Competing Risk of All-Cause Death

Outcomes	Finerenone vs. Placebo Subdistribution HR (95% CI)
**Cardiovascular events[a]**	
Cardiovascular death or heart failure hospitalization	0.83 (0.74, 0.93)
Cardiovascular death	0.84 (0.71, 0.998)
Heart failure hospitalization	0.81 (0.71, 0.93)
Major adverse cardiovascular events	0.90 (0.83, 0.99)
New-onset atrial fibrillation	0.83 (0.70, 0.99)
**Cardiovascular events[b]**	
Cardiovascular death or heart failure hospitalization	0.85 (0.77, 0.94)
Cardiovascular death	0.87 (0.77, 0.999)
Major adverse cardiovascular events	0.90 (0.82, 0.97)
**Kidney outcomes**	
Composite kidney outcome (eGFR ≥50%)[c]	0.80 (0.71, 0.89)
Composite kidney outcome (eGFR ≥57%)[d]	0.79 (0.69, 0.90)
**All-cause morbidity**	
All-cause hospitalization	0.97 (0.93, 1.02)

[a]: Endpoints inclusive of cardiovascular death exclusive of deaths with undetermined causes.
[b]: Endpoints inclusive of cardiovascular death inclusive of deaths with undetermined causes.
[c]: Reflects time to first sustained decrease in estimated glomerular filtration rate to ≥50% from baseline, sustained decrease in estimated glomerular filtration rate to <15 mL/min/1.73 m^2, kidney failure and death due to kidney failure.
[d]: Reflects time to first sustained decrease in estimated glomerular filtration rate to ≥57% from baseline, sustained decrease in estimated glomerular filtration rate to <15 mL/min/1.73 m^2, kidney failure and death due to kidney failure.
Major adverse cardiovascular events reflects the composite of non-fatal myocardial infarction, non-fatal stroke, heart failure hospitalization, and cardiovascular death.
Subdistribution hazard ratios and 95% CI estimated through Fine-Gray methods (competing risk = all-cause mortality), adjusting for trial and geographic region.
Abbreviations: eGFR = estimated glomerular filtration rate; HR = hazard ratio; py = person-years

Supplemental Table 5: Treatment Effects of Finerenone vs. Placebo on Morbidity and Mortality in a Sensitivity Analysis Including FIDELIO-DKD, FIGARO-DKD, and FINEARTS-HF Participants with Type 2 Diabetes and At Least Moderate-Risk Chronic Kidney Disease

	Finerenone (n=7354)		Placebo (n=7346)		Finerenone vs. Placebo
**Outcomes**	**# with Event (%)**	**Incidence Rate (per 1000 py)**	**# with Event (%)**	**Incidence Rate (per 1000 py)**	**HR (95% CI)**
**Cardiovascular events**[a]					
Cardiovascular death or HF hospitalization	646 (9)	30.4	750 (10)	35.7	0.86 (0.77, 0.95)
Cardiovascular death	273 (4)	12.5	311 (4)	14.3	0.87 (0.74, 1.03)
HF hospitalization	434 (6)	20.4	521 (7)	24.8	0.83 (0.73, 0.94)
Major adverse cardiovascular events	995 (14)	48.2	1082 (15)	53.0	0.92 (0.84, 1.00)
New-onset atrial fibrillation	235 (4)[b]	12.5	279 (4)[b]	14.9	0.84 (0.70, 1.00)
**Cardiovascular events**[c]					
Cardiovascular death or HF hospitalization	797 (11)	37.6	909 (12)	43.2	0.87 (0.79, 0.96)
Cardiovascular death	446 (6)	20.4	499 (7)	22.9	0.89 (0.78, 1.01)
Major adverse cardiovascular events	1122 (15)	54.4	1233 (17)	60.4	0.90 (0.83, 0.98)
**Kidney outcomes**					
Composite kidney outcome (eGFR ≥50%)[d]	517 (7)	26.1	651 (9)	33.1	0.78 (0.70, 0.88)
Composite kidney outcome (eGFR ≥57%)[e]	375 (5)	18.7	479 (7)	24.1	0.78 (0.68, 0.89)
**All-cause morbidity and mortality**					
All-cause death	746 (10)	34.1	806 (11)	37.0	0.92 (0.83, 1.01)
All-cause hospitalization	3313 (45)	205.0	3403 (46)	213.1	0.96 (0.92, 1.01)
All-cause death and all-cause hospitalization	3460 (47)	214.1	3581 (49)	224.3	0.96 (0.91, 1.00)

[a]: Endpoints inclusive of cardiovascular death exclusive of deaths with undetermined causes.
[b]: Percentage displayed out of participants without atrial fibrillation at baseline.
[c]: Endpoints inclusive of cardiovascular death inclusive of deaths with undetermined causes.
[d]: Reflects time to first sustained decrease in estimated glomerular filtration rate to ≥50% from baseline, sustained decrease in estimated glomerular filtration rate to <15 mL/min/1.73 m^2, kidney failure and death due to kidney failure.
[e]: Reflects time to first sustained decrease in estimated glomerular filtration rate to ≥57% from baseline, sustained decrease in estimated glomerular filtration rate to <15 mL/min/1.73 m^2, kidney failure and death due to kidney failure.
Major adverse cardiovascular events reflects the composite of non-fatal myocardial infarction, non-fatal stroke, HF hospitalization, and cardiovascular death.
Abbreviations: eGFR = estimated glomerular filtration rate; HF = heart failure; HR = hazard ratio; IR = incidence rate; py = person-years

Supplemental Table 6: Treatment Effects of Finerenone vs. Placebo on Morbidity and Mortality in a Sensitivity Analysis Including FIDELIO-DKD, FIGARO-DKD, and FINEARTS-HF Participants with eGFR >25 mL/min/1.73 m^2 and UACR >30 mg/g, With or Without Type 2 Diabetes

Outcomes	Finerenone (n=7602)		Placebo (n=7610)		Finerenone vs. Placebo
	# with Event (%)	Incidence Rate (per 1000 py)	# with Event (%)	Incidence Rate (per 1000 py)	HR (95% CI)
**Cardiovascular events[a]**					
Cardiovascular death or HF hospitalization	709 (9)	32.6	843 (11)	39.1	0.84 (0.76, 0.93)
Cardiovascular death	294 (4)	13.1	347 (5)	15.5	0.85 (0.73, 0.99)
HF hospitalization	490 (6)	22.5	590 (8)	27.4	0.83 (0.74, 0.94)
Major adverse cardiovascular events	1069 (14)	50.6	1181 (16)	56.4	0.90 (0.83, 0.98)
New-onset atrial fibrillation	242 (4)[b]	12.9	295 (5)[b]	15.7	0.83 (0.70, 0.98)
**Cardiovascular events[c]**					
Cardiovascular death or HF hospitalization	866 (11)	39.8	1002 (13)	46.5	0.86 (0.78, 0.94)
Cardiovascular death	472 (6)	21.0	542 (7)	24.2	0.87 (0.77, 0.98)
Major adverse cardiovascular events	1200 (16)	56.8	1332 (18)	63.6	0.90 (0.83, 0.97)
**Kidney outcomes**					
Composite kidney outcome (eGFR ≥50%)[d]	530 (7)	26.1	657 (9)	32.5	0.80 (0.71, 0.89)
Composite kidney outcome (eGFR ≥57%)[e]	384 (5)	18.7	487 (6)	23.9	0.78 (0.69, 0.90)
**All-cause morbidity and mortality**					
All-cause death	785 (10)	34.9	867 (11)	38.7	0.90 (0.82, 1.00)
All-cause hospitalization	3444 (45)	207.7	3564 (47)	217.6	0.96 (0.91, 1.00)
All-cause death and all-cause hospitalization	3591 (47)	216.6	3751 (49)	229.0	0.95 (0.91, 0.99)

[a]: Endpoints inclusive of cardiovascular death exclusive of deaths with undetermined causes.
[b]: Percentage displayed out of participants without atrial fibrillation at baseline.
[c]: Endpoints inclusive of cardiovascular death inclusive of deaths with undetermined causes.
[d]: Reflects time to first sustained decrease in estimated glomerular filtration rate to ≥50% from baseline, sustained decrease in estimated glomerular filtration rate to <15 mL/min/1.73 m^2, kidney failure and death due to kidney failure.
[e]: Reflects time to first sustained decrease in estimated glomerular filtration rate to ≥57% from baseline, sustained decrease in estimated glomerular filtration rate to <15 mL/min/1.73 m^2, kidney failure and death due to kidney failure.
Major adverse cardiovascular events reflects the composite of non-fatal myocardial infarction, non-fatal stroke, HF hospitalization, and cardiovascular death.
Abbreviations: eGFR = estimated glomerular filtration rate; HF = heart failure; HR = hazard ratio; IR = incidence rate; py = person-years

Supplemental Table 7: Incidence of Hyperkalemia and Hypokalemia, According to Baseline eGFR Category

eGFR Category (mL/min/1.73 m^2)	n	All Participants	Finerenone	Placebo
**Any serum potassium >5.5 mEq/L[a]**				
≥90	1411	88 (6%)	55 (8%)	33 (5%)
≥60 to <90	4233	421 (10%)	288 (14%)	133 (6%)
≥45 to <60	3756	453 (12%)	307 (17%)	146 (8%)
≥30 to <45	3810	662 (18%)	472 (25%)	190 (10%)
<30	942	174 (19%)	110 (24%)	64 (13%)
**Any serum potassium >6.0 mEq/L[a]**				
≥90	1411	10 (1%)	3 (0.4%)	7 (1%)
≥60 to <90	4233	90 (2%)	63 (3%)	27 (1%)
≥45 to <60	3756	75 (2%)	55 (3%)	20 (1%)
≥30 to <45	3810	141 (4%)	102 (5%)	39 (2%)
<30	942	37 (4%)	29 (6%)	8 (2%)
**Hyperkalemia leading to hospitalization**				
≥90	1411	0 (0%)	0 (0%)	0 (0%)
≥60 to <90	4233	10 (0.2%)	10 (1%)	0 (0%)
≥45 to <60	3756	22 (1%)	17 (1%)	5 (0.3%)
≥30 to <45	3810	32 (1%)	28 (2%)	4 (0.2%)
<30	942	20 (2%)	14 (3%)	6 (1%)
**Any serum potassium <3.5 mEq/L[a]**				
≥90	1411	112 (8%)	37 (5%)	75 (11%)
≥60 to <90	4233	308 (7%)	106 (5%)	202 (10%)
≥45 to <60	3756	281 (8%)	77 (4%)	204 (11%)
≥30 to <45	3810	267 (7%)	97 (5%)	170 (9%)
<30	942	81 (9%)	21 (5%)	60 (13%)

[a]: Based on central laboratory measurements of potassium levels
Abbreviations: eGFR = estimated glomerular filtration rate

Supplemental Figure 1: Distribution of Kidney Risk in FINE-HEART Participants with Chronic Kidney Disease and Type 2 Diabetes

		UACR (mg/g)		
		A1	A2	A3
	eGFR (mL/min/1.73 m^2)	<30	30-300	>300
G1	≥90	13 (0.1%)	269 (1.9%)	1132 (8.0%)
G2	60-89	51 (0.4%)	1311 (9.2%)	2875 (20.3%)
G3a	45-59	82 (0.6%)	1610 (11.4%)	2070 (14.6%)
G3b	30-44	68 (0.5%)	1429 (10.1%)	2324 (16.4%)
G4	15-29	16 (0.1%)	271 (1.9%)	659 (4.6%)
G5	<15	0 (0.0%)	0 (0.0%)	0 (0.0%)

KDIGO Risk Categories			
Low	Moderate	High	Very High
0.5%	11.7%	40.1%	47.7%

Distribution of kidney risk categories according to eGFR and UACR among FINE-HEART participants with CKD and T2D included in the analysis.
Abbreviations: CKD = chronic kidney disease; eGFR = estimated glomerular filtration rate; KDIGO = Kidney Disease: Improving Global Outcomes; T2D = type 2 diabetes; UACR = urine albumin-to-creatinine ratio

Supplemental Figure 2: Treatment Effects of Finerenone vs. Placebo on the Composite Kidney Outcome (eGFR ≥57%) Among FINE-HEART Participants with Chronic Kidney Disease and Type 2 Diabetes, According to Baseline eGFR, UACR, and HbA$_{1c}$

A: Baseline eGFR **B: Baseline UACR** **C: Baseline HbA$_{1c}$**

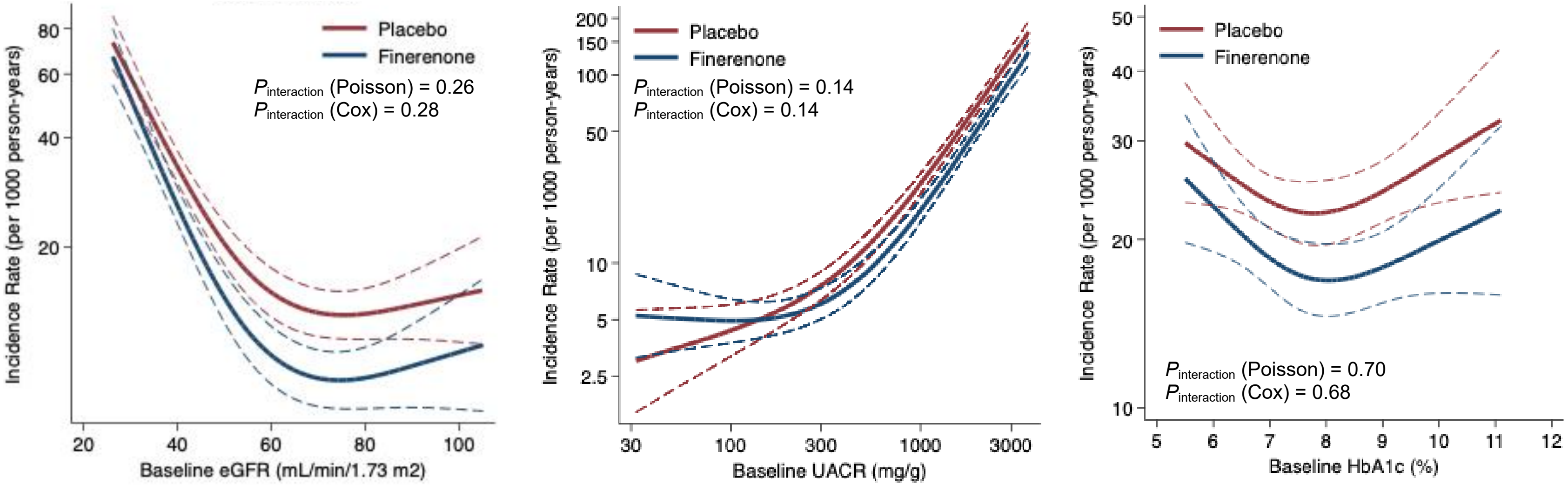

Figure shows the incidence rate per 1,000 person-years (and 95% CI), estimated through Poisson regression, of the composite kidney outcome inclusive of a ≥57% eGFR decline from baseline, according to baseline eGFR, UACR, and HbA$_{1c}$. Treatment effect modification evaluated through Poisson regression and Cox proportional hazards regression, in both cases with restricted cubic splines with 3 knots. Comparisons reflect use of pooled individual-level data.

Abbreviations: eGFR = estimated glomerular filtration rate; HbA$_{1c}$ = glycated hemoglobin; UACR = urine albumin-to-creatinine ratio

Supplemental Figure 3: Effect of Finerenone on the Composite Kidney Outcome in FINE-HEART Participants with Chronic Kidney Disease and Type 2 Diabetes, Overall and in Key Subgroups

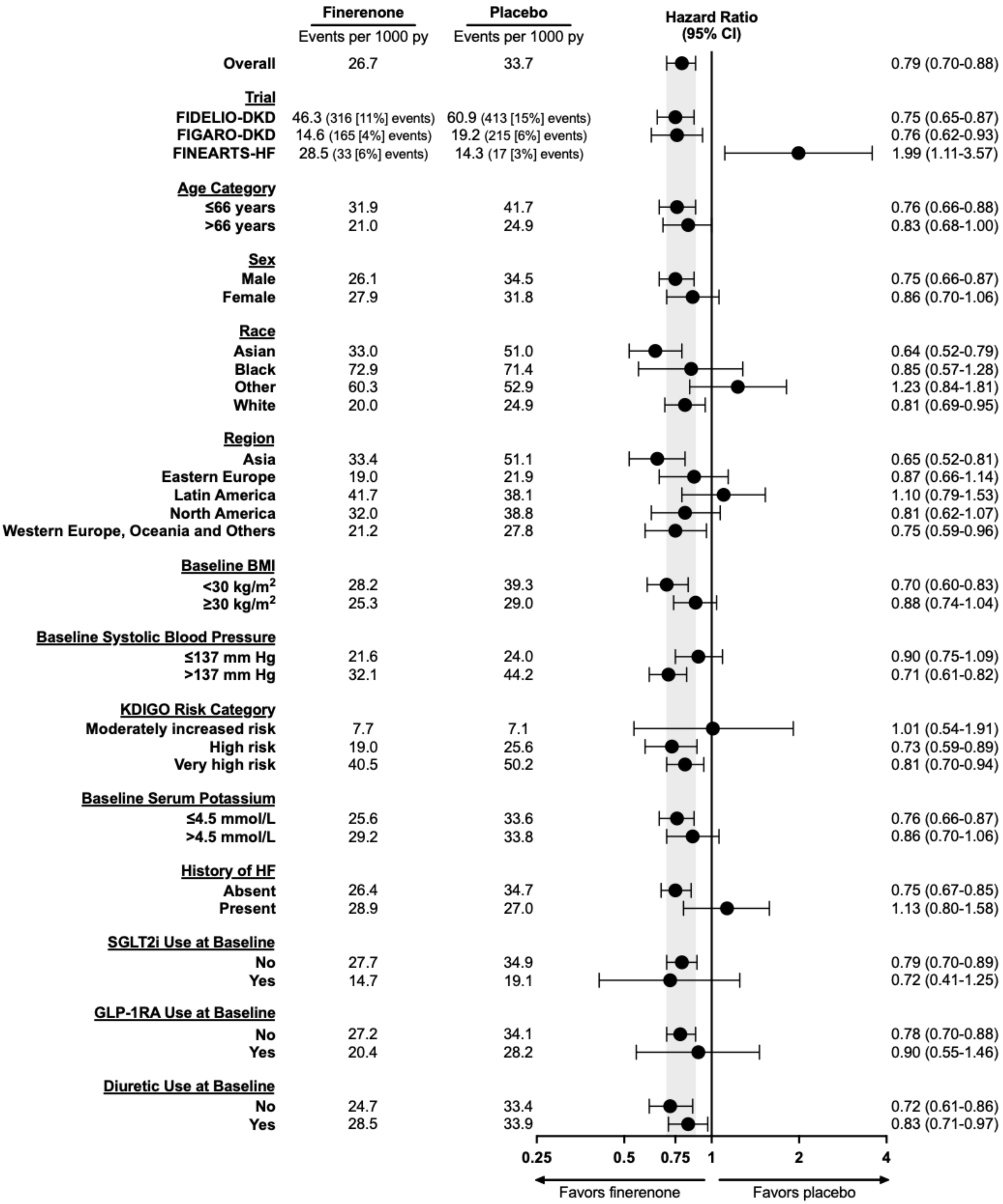

Forest plot shows treatment effects of finerenone versus placebo on the composite kidney outcome (eGFR ≥50% decrease), overall and in key subgroups.
Abbreviations: BMI = body mass index; CV = cardiovascular; GLP-1RA = glucagon-like peptide-1 receptor agonist; HF = heart failure; KDIGO = Kidney Disease: Improving Global Outcomes; py = person-years; SGLT2i = sodium-glucose co-transporter 2 inhibitor

Supplemental Figure 4: Treatment Effects of Finerenone vs. Placebo on Cardiovascular and Kidney Outcomes in a Sensitivity Analysis Including FIDELIO-DKD, FIGARO-DKD, and FINEARTS-HF Participants with Type 2 Diabetes and At Least Moderate-Risk Chronic Kidney Disease, According to Baseline eGFR and UACR

A: CV Death and HF Hospitalization, by Baseline eGFR

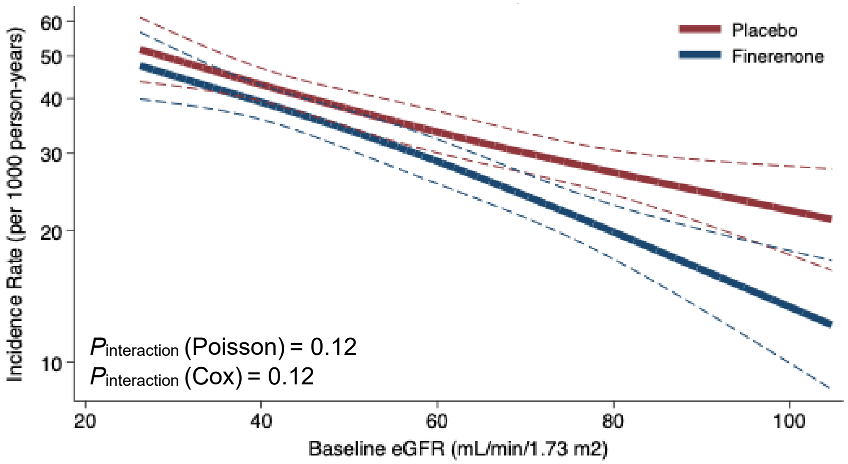

B: CV Death and HF Hospitalization, by Baseline UACR

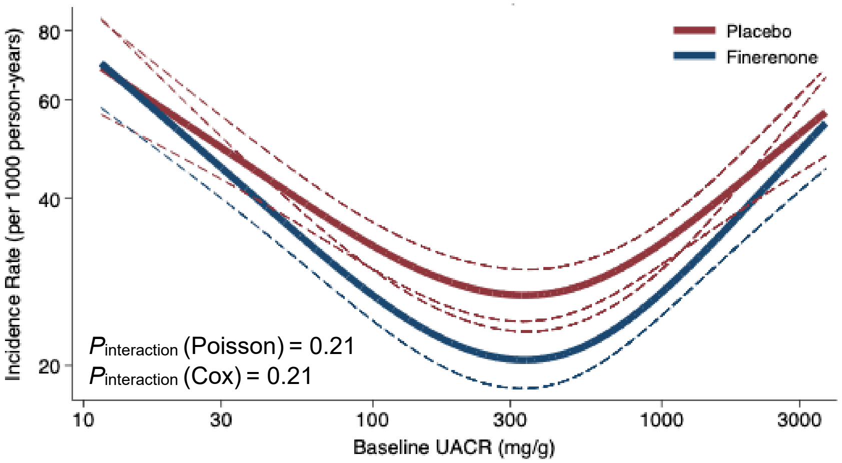

C: Kidney Composite[a], by Baseline eGFR

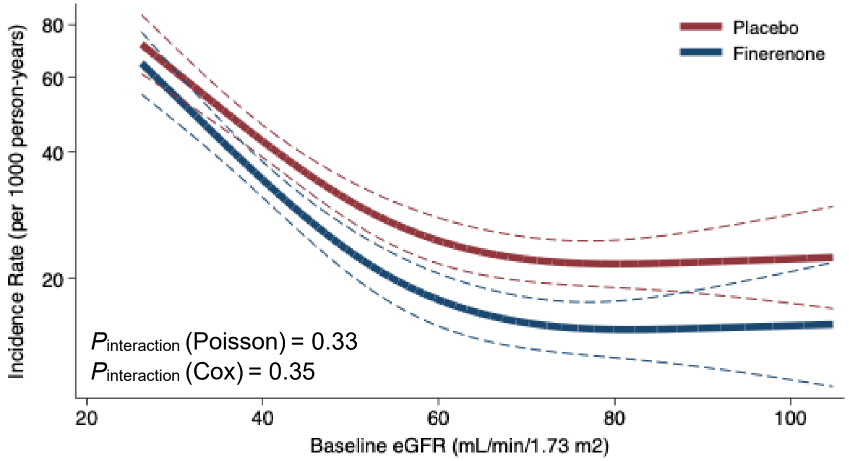

D: Kidney Composite[a], by Baseline UACR

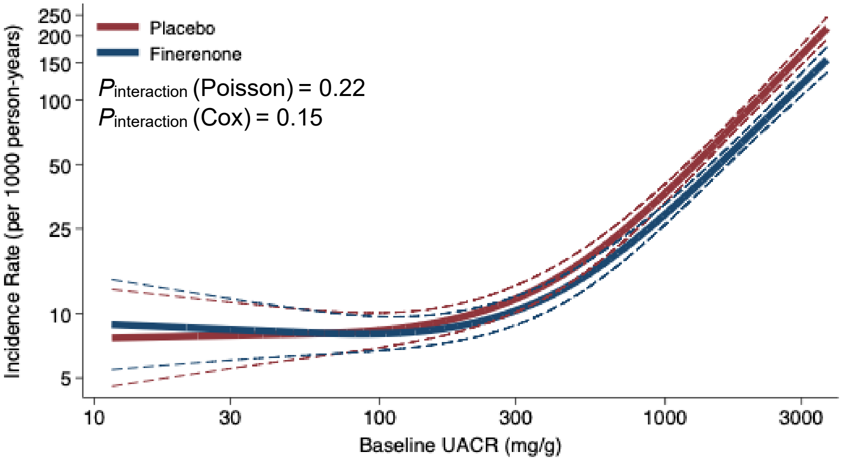

[a]: Time to first sustained decrease in eGFR to ≥50% from baseline, sustained decrease in estimated glomerular filtration rate to <15 mL/min/1.73 m^2, kidney failure and death due to kidney failure.
Figure shows treatment effects (with 95% CI) of finerenone vs. placebo on CV death and HF hospitalization according to baseline eGFR (A) and UACR (B), estimated through Poisson regression and displayed as restricted cubic splines with 3 knots. CV death is exclusive of deaths with undetermined causes.
Abbreviations: CV = cardiovascular; eGFR = estimated glomerular filtration rate; HF = heart failure; UACR = urine albumin-to-creatinine ratio

Supplemental Figure 5: Treatment Effects of Finerenone vs. Placebo on Cardiovascular and Kidney Outcomes in a Sensitivity Analysis Including FIDELIO-DKD, FIGARO-DKD, and FINEARTS-HF Participants with eGFR >25 mL/min/1.73 m^2 and UACR >30 mg/g, With or Without Type 2 Diabetes, According to Baseline eGFR, UACR, and HbA$_{1c}$

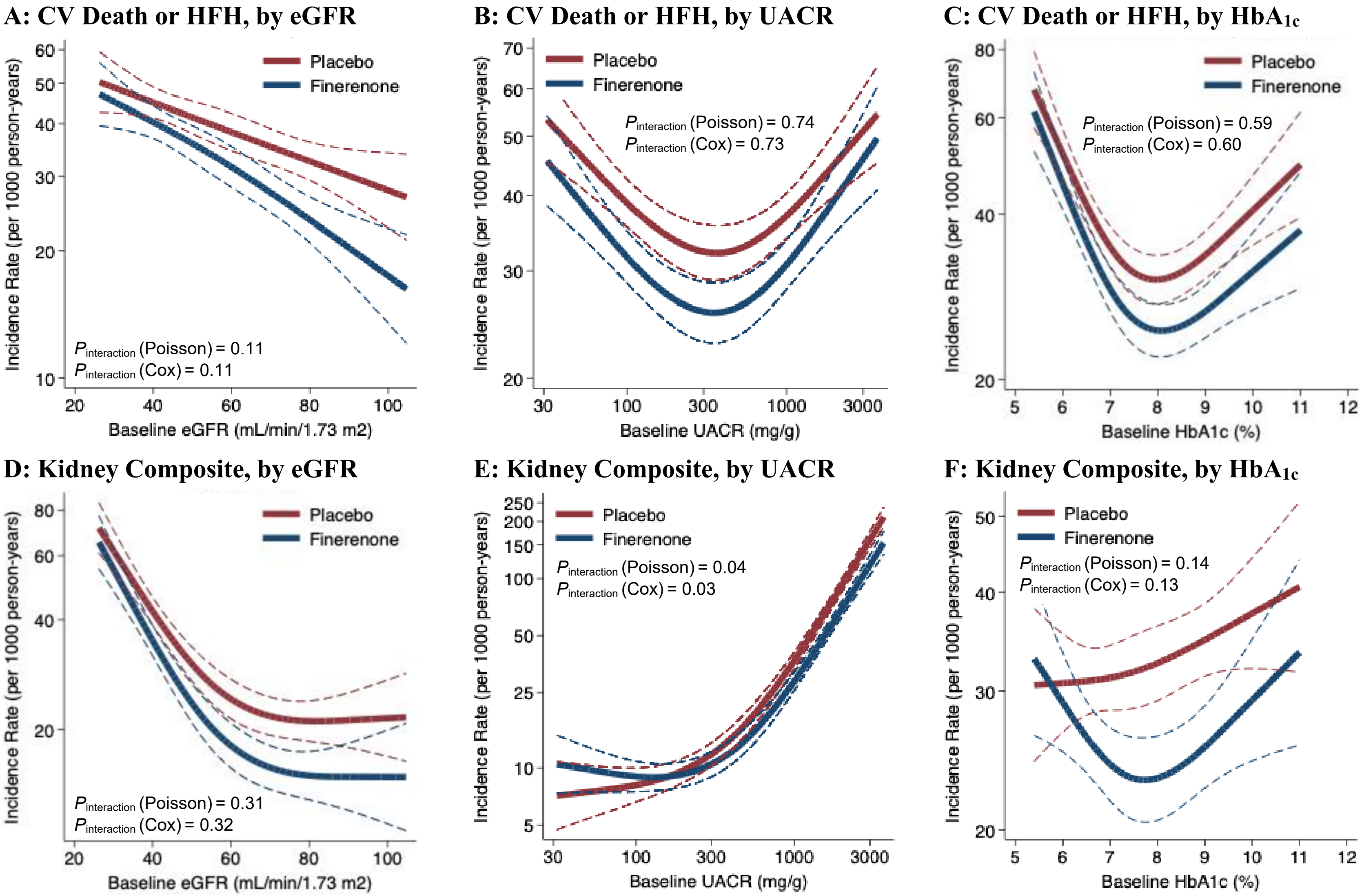

Figure shows incidence rates per 1,000 person-years (with 95% CI), estimated through Poisson regression, by treatment arm for CV death and HFH (A-C) and the composite kidney outcome inclusive of ≥50% eGFR decline (D-F) according to baseline eGFR (A and D), UACR (B and E), and HbA$_{1c}$ (C and F). Treatment effect modification evaluated using Poisson regression and Cox proportional hazards regression models with restricted cubic splines (3 knots). CV death is exclusive of deaths with undetermined causes.

Abbreviations: CV = cardiovascular; eGFR = estimated glomerular filtration rate; HbA$_{1c}$ = glycated hemoglobin; HFH = heart failure hospitalization; UACR = urine albumin-to-creatinine ratio
